# Stereoselective Synthesis, Synthetic and Pharmacological Application of Monoterpene-Based 1,2,4- and 1,3,4-Oxadiazoles

**DOI:** 10.3390/ijms19010081

**Published:** 2017-12-28

**Authors:** Tímea Gonda, Péter Bérdi, István Zupkó, Ferenc Fülöp, Zsolt Szakonyi

**Affiliations:** 1 Institute of Pharmaceutical Chemistry, University of Szeged, H-6720 Szeged, Eötvös utca 6, Hungary; gonda.timea@pharm.u-szeged.hu (T.G.); fulop@pharm.u-szeged.hu (F.F.); 2 Department of Pharmacodynamics and Biopharmacy, University of Szeged, H-6720 Szeged, Eötvös utca 6, Hungary; berdipeter@gmail.com (P.B.); zupko@pharm.u-szeged.hu (I.Z.); 3 Interdisciplinary Centre of Natural Products, University of Szeged, H-6720 Szeged, Eötvös utca 6, Hungary; 4 Stereochemistry Research Group of the Hungarian Academy of Sciences, H-6720 Szeged, Eötvös utca 6, Hungary

**Keywords:** terpenoid, stereoselective, 1,2,4-oxadiazole, 1,3,4-oxadiazole, chiral catalyst, diethyl zinc, antiproliferative activity

## Abstract

Stereoselective synthesis of monoterpene-based 1,2,4- and 1,3,4-oxadiazole derivatives was accomplished starting from α,β-unsaturated carboxylic acids, obtained by the oxidation of (−)-2-carene-3-aldehyde and commercially available (−)-myrtenal. 1,2,4-Oxadiazoles were prepared in two steps via the corresponding *O*-acylamidoxime intermediates, which then underwent cyclisation induced by tetrabutylammonium fluoride (TBAF) under mild reaction conditions. Stereoselective dihydroxylation in highly stereospecific reactions with the OsO_4_/NMO (*N*-methylmorpholine *N*-oxide) system produced α,β-dihydroxy 1,2,4-oxadiazoles. Pinane-based 1,3,4-oxadiazoles were obtained similarly from acids by coupling with acyl hydrazines followed by POCl_3_-mediated dehydrative ring closure. In the case of the arane counterpart, the rearrangement of the constrained carane system occurred with the loss of chirality under the same conditions. Stereoselective dihydroxylation with OsO_4_/NMO produced α,β-dihydroxy 1,3,4-oxadiazoles. The prepared diols were applied as chiral catalysts in the enantioselective addition of diethylzinc to aldehydes. All compounds were screened in vitro for their antiproliferative effects against four malignant human adherent cell lines by means of the MTT assay with the *O*-acylated amidoxime intermediates exerting remarkable antiproliferative action.

## 1. Introduction

In asymmetric synthetic chemistry a growing demand occurs for new chiral ligands and synthons. New strategies are being developed for the synthesis of reliable enantiopure catalysts [1,2,3]. Incorporation of chirality into ligands by applying optically active monoterpenes as starting materials bears several advantages: the natural chiral origin can determine the stereochemistry of newly forming chiral centers as well the chiral activities of these newly desired chiral catalysts [4,5]. A large variety of chiral amino alcohol and aminodiol-type ligands, derived from monoterpenes, such as α- and β-pinene [6,7,8,9,10], (−)-(*S*)-perillaldehyde, carene [11,12,13], and fenchone-camphor [13,14], have been reported as successful chiral catalysts. We recently described the synthesis and transformation of enantiomerically pure pinane- and carane-based 3-amino-1,2-diols, whereas the amino moiety served as a fundamental building block in the synthesis of terpenoid-type nucleoside analogues with remarkable sodium/calcium exchanger (NCX) inhibitor activity [15,16]. Monoterpene-based 1,3-heterocycles, such as 1,3-oxazines or -oxazolidines could be successfully applied as chiral catalysts in a wide range of stereoselective transformations [11,17,18,19,20,21].

On the other hand, 1,2,4- and 1,3,4-oxadiazoles have also been extensively studied in the past decade [22,23,24,25,26,27,28]. A wide range of biologically active pharmacophores possess these five-membered heteroaromatic ring systems with remarkable biological properties including sphinosine kinase inhibitor [29], diacylglycerol acyltransferase 1 (DGAT-1) inhibitor [30], glycogen synthase kinase (GSK-3) inhibitor [31], sirtuin (SIRT) inhibitor [32] and methionine aminopeptidase inhibitor activities [33]. Some diterpenic 1,2,4- and 1,3,4-oxadiazoles, including steroid-based compounds, have also shown remarkable antiproliferative action on adherent human cancer cell lines [25,26,34,35,36,37].

In the present work, we set out to create pinane- and carane-based dihydroxy-derived 1,2,4- and 1,3,4-oxadiazoles as heterocyclic analogues of previously prepared monoterpenic 3-amino-1,2-diol library. The syntheses were started from readily available (−)-(*S*)-perillaldehyde and (−)-myrtenal, as natural monoterpene sources, then applying the resulting tridental ligands as chiral catalysts in the enantioselective addition of diethylzinc to benzaldehyde. We also planned to screen the prepared compounds in vitro for their antiproliferative effects against four malignant human adherent cell lines by means of the MTT [3-(4,5-dimethylthiazol-2-yl)-2,5-diphenyltetrazolium bromide] assay.

## 2. Results

### 2.1. Synthesis of Monoterpene-Based 1,2,4- and 1,3,4-Oxadiazoles

**Scheme 1:**
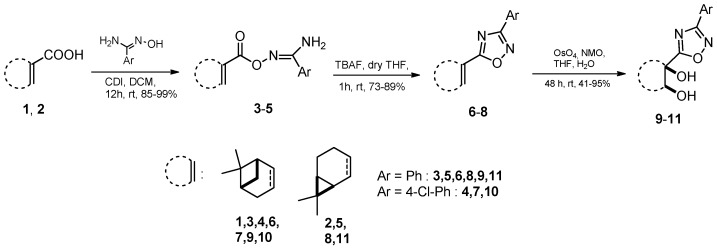
Stereoselective synthesis of monoterpene-based 1,2,4-oxadiazoles. TBAF: tetrabutylammonium fluoride; THF: tetrahydrofurane; CDI: 1,1′-carbonyldiimidazole; DCM: dichloromethane.

Stereoselective synthesis of monoterpene-based 1,2,4-oxadiazole derivatives was accomplished starting from α,β-unsaturated carboxylic acids prepared previously through the oxidation of (−)-2-carene-3-aldehyde (derived in a two-step reaction from (−)-(*S*)-perillaldehyde) using commercially available (−)-myrtenal [12,38]. There are several methods for the preparation of 1,2,4-oxazoles starting from compounds bearing carboxyl function. Because of the well-known sensitivity of constrained bicyclic monoterpenes, however, a pathway with mild reaction condition was applied in our case [26]. Carboxylic acids **1** and **2** were coupled with amidoximes in the presence of CDI in DCM with excellent yields, followed by TBAF-catalyzed ring closure of *O*-acylated amidoximes **3**–**5** at room temperature. The obtained α,β-unsaturated 3-aryl-1,2,4-oxazoles **6**–**8** were stereoselectively dihydroxylated with the OsO_4_/NMO system resulting in **9**–**11** as single diastereoisomers (Scheme 1) [8,20]. The configuration of the new stereogenic centers was determined by NMR (nuclear magnetic resonance) with Nuclear Overhauser Effect Spectroscopy (NOESY) experiments.

**Scheme 2:**
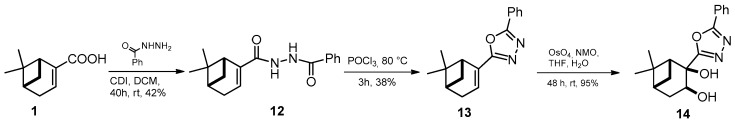
Stereoselective synthesis of pinane-based 1,3,4-oxadiazole **14**.

The synthesis of the isomeric 1,3,4-oxadiazole ring system was also started from monoterpenic acids **1** and **2**. In this case, however, we have found different reactivity between the pinane and carane ring system under the ring-closure process. Myrtenic acid **1** was coupled with benzhydrazide in the presence of CDI. Cyclodehydration of *N*,*N*′-diacylhydrazine **12** with POCl_3_ at 80 °C furnished **13** with moderate yield [25]. Dihydroxylation of **13** with OsO_4_/NMO afforded **14** in a highly diastereoselective reaction, similar to 1,2,4-oxadiazoles **6**–**8** (Scheme 2).

**Scheme 3:**
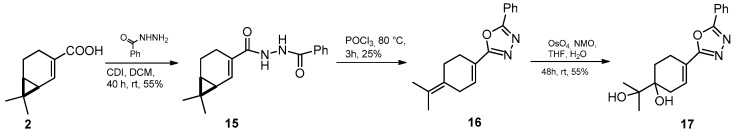
Rearrangement of the carane ring towards cycloxene-based 1,3,4-oxadiazoles.

Starting from (−)-2-carene-3-carboxylic acid **2**, subsequent ring closure of *N*,*N*′-diacylhydrazine **15** resulted in 1,3,4-oxazole **16** with ring rearrangement and loss of chirality of the carane ring system (Scheme 3). Dihydroxylation of **16** under mild conditions provided *exo*-dihydroxylated racemic oxadiazole **17** with moderate yield.

### 2.2. Application of the Prepared Catalysts ***9***–***11*** and ***14***

**Scheme 4:**
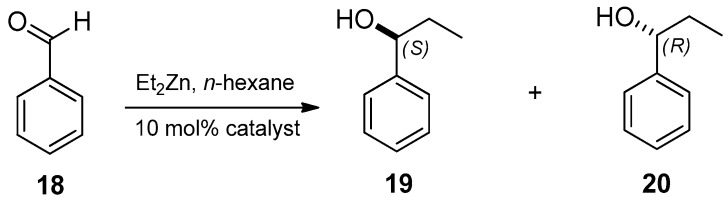
Model reaction for enantioselective catalysis.

The prepared potential catalysts **9**–**11** and **14** were used in the reaction of diethylzinc and benzaldehyde affording the formation of chiral 1-phenyl-1-propanol as a reference product (Scheme 4). They were applied in a 10% molar ratio in *n*-hexane at room temperature. The enantiomeric purities of 1-phenyl-1-propanols **19** and **20** obtained were determined by GC on a Chirasil-DEX CB column, according to literature methods [39,40]. Our results are presented in Table 1.

Moderate enantioselectivity was observed for carane-based 1,2,4-oxadiazole **11** with *S* selectivity, while pinane-based 1,2,4-oxadiazoles (**9** and **10**) and 1,3,4-oxadiazole **14** have shown reasonable, similar *S* selectivity (up to 74% *ee*, Table 1) in the model reaction. We found that the type of the heterocyclic ring system has affected catalytic activities.

### 2.3. Antiproliferative Activities

Since several steroid-based 1,2,4- and 1,3,4-oxadiazoles as well as their amidoxime type intermediates have shown exerting antiproliferative action on adherent human cancer cell lines [25,26,34], antiproliferative activities of the prepared analogues were also tested against a panel of human malignant cell lines isolated from cervical (HeLa), ovarian (A2780), and breast (MCF7 and MDA-MB-231) cancers (Table 2). *O*-Acylated amidoximes (**3**–**5**) exhibited remarkable growth inhibitory activities with calculated IC_50_ values comparable to those of reference agent cisplatin. Compounds with pinane building block (**3** and **4**) were slightly more effective than their carane containing analog (**5**) and A2780 cells seemed to be outstandingly sensitive (IC_50_ values: 1.44–2.05 μM). These active molecules displayed moderately lower antiproliferative activities against triple-negative breast cancer cell line (MDA-MB-231) than other utilized gynecological cell lines. All prepared and tested oxadiazoles (**6**–**14**), with the exception of compound **6**, exerted substantially lower action at 10 and 30 μM against A2780 cells. Therefore, no additional experiments were carried out in order to calculate their IC_50_ values.

## 3. Discussion

Starting from monoterpenic acids, dihydroxy-substituted 1,2,4-oxadiazoles and 1,3,4-oxadiazole were prepared and their catalytic activities were examined in the enantioselective synthesis of 1-phenyl-1-propanol. In pharmacological studies the *O*-acylamidoxime intermediates showed remarkable cytotoxic activity. Under the construction of the 1,3,4-oxadiazole system, rearrangement of the carane ring was observed with loss of chirality.

## 4. Materials and Methods

### 4.1. General Methods

Commercially available compounds were used as obtained from suppliers (Molar Chemicals Ltd, Halásztelek, Hungary; Merck Ltd., Budapest, Hungary and VWR International Ltd., Debrecen, Hungary) while applied solvents were dried according to standard procedures. Optical rotations were measured in MeOH at 20 °C, with a Perkin-Elmer 341 polarimeter (PerkinElmer Inc., Shelton, CT, USA). Chromatographic separations and monitoring of reactions were carried out on Merck Kieselgel 60 (Merck Ltd., Budapest, Hungary). Elemental analyses for all prepared compounds were performed on a Perkin-Elmer 2400 Elemental Analyzer (PerkinElmer Inc., Waltham, MA, USA). GC measurements for direct separation of enantiomers was performed on a Chirasil-DEX CB column (2500 × 0.25 mm I.D.) on a Perkin-Elmer Autosystem XL GC consisting of a Flame Ionization Detector (Perkin-Elmer Corporation, Norwalk, CT, USA) and a Turbochrom Workstation data system (Perkin-Elmer Corp., Norwalk, CT, USA). Melting points were determined on a Kofler apparatus (Nagema, Dresden, Germany) and are uncorrected. ^1^H and ^13^C NMR spectroscopic data were recorded at room temperature on a Bruker Avance DRX 400 MHz spectrometer (Bruker Corp., Billerica, MA, USA) in CDCl_3_ or in DMSO.

### 4.2. General Procedure for the Preparation of ***3***, ***4*** and ***5***

**1** or **2** (6.02 mmol) was dissolved in dry CH_2_Cl_2_ (75 mL) and CDI (9.07 mmol) was added. The solution was stirred at room temperature for 2 h, then benzamid oxime (18.07 mmol) or 4-chlorobenzamide oxime (18.07 mmol) was added in one portion. The mixture was stirred overnight then evaporated to dryness and purified by column chromatography on silica gel with CH_2_Cl_2_/EtOAc 19:1.

*(E)-N′-(((1R,5S)-6,6-dimethylbicyclo[3.1.1]hept-2-ene-2-carbonyl)oxy)benzimidamide* (**3**): Yield: 85%, white crystals, m.p.: 133–136 °C. [α]${}_{D}^{20}$ = −40 (c 0.250, MeOH). ^1^H NMR (400 MHz, dimethyl sulfoxide (DMSO)): δ 7.77–7.71 (2H, m), 7.54–7.42 (3H, m), 7.10–7.06 (1H, m), 2.80 (1H, dt, *J* = 1.3 Hz, 5.6 Hz), 2.49–2.36 (3H, m), 2.17–2.11 (1H, m), 1.34 (3H, s), 1.10 (1H, d, *J* = 8.8 Hz), 0.80 (3H, s). ^13^C NMR (100 MHz, DMSO): δ 162.9, 156.2, 138.3, 136.2, 131.9, 130.3, 128.2, 126.8, 40.8, 39.8, 37.2, 31.7, 30.9, 25.7, 20.8 (Figure S1). Anal. calcd. for C_17_H_20_N_2_O_2_: C, 71.81; H, 7.09; N, 9.85. Found: C, 71.68; H, 7.13; N, 9.81.

*(E)-4-chloro-N′-(((1R,5S)-6,6-dimethylbicyclo[3.1.1]hept-2-ene-2-carbonyl)oxy)benzimidamide* (**4**): Yield: 99%, white crystals, m.p.: 144–146 °C. [α]${}_{D}^{20}$ = −30 (c 0.250, MeOH). ^1^H NMR (400 MHz, DMSO): δ 7.74 (2H, d, *J* = 8.6 Hz), 7.52 (2H, d, *J* = 8.6 Hz), 7.10–7.06 (1H, m), 6.77 (2H, br s), 2.78 (1H, dt, *J* = 1 Hz, 5.7 Hz), 2.48–2.44 (2H, m), 2.41 (1H, t, *J* = 2.9 Hz), 2.15–2.09 (1H, m), 1.33 (3H, s), 1.09 (1H, d, *J* = 8.9 Hz), 0.79 (3H, s). ^13^C NMR (100 MHz, DMSO): δ 162.8, 155.2, 138.2, 136.4, 135.0, 130.7, 128.6, 128.3, 40.8, 39.8, 37.2, 31.7, 30.9, 25.7, 20.8 (Figure S2). Anal. calcd. for C_17_H_19_ClN_2_O_2_: C, 64.05; H, 6.01; N, 8.79. Found: C, 64.11; H, 6.09; N, 8.59.

*N′-(((1R,6S)-7,7-dimethylbicyclo[4.1.0]hept-2-ene-3-carbonyl)oxy)benzimidamide* (**5**): Yield: 87%, colorless oil. [α]${}_{D}^{20}$ = +129 (c 0.250, MeOH). ^1^H NMR (400 MHz, DMSO): δ 7.74–7.69 (2H, m), 7.52–7.37 (4H, m), 6.67 (2H, br s), 2.47–2.38 (1H, m), 2.00–1.82 (2H, m), 1.81–1.70 (1H, m), 1.36–1.31 (1H, m), 1.21–1.17 (1H, m), 1.16 (3H, s), 0.92 (3H, s). ^13^C NMR (100 MHz, DMSO): δ 164.0, 156.3, 138.9, 131.9, 130.3, 128.2, 126.7, 126.1, 28.8, 28.4, 24.9, 23.4, 21.3, 16.6, 15.7 (Figure S3). Anal. calcd. for C_17_H_20_N_2_O_2_: C, 71.81; H, 7.09; N, 9.85. Found: C, 71.88; H, 7.03; N, 9.88.

### 4.3. General Procedure for the Preparation of ***6***, ***7*** and ***8***

**3**, **4** or **5** (3.52 mmol), was dissolved in freshly-distilled THF (35 mL) and TBAF (1 M solution in THF, 0.35 mL) was added under Ar atmosphere. The solution was stirred for 1 h then water (50 mL) was added and the mixture was extracted with CH_2_Cl_2_ (3 × 80 mL). The organic phase was dried (Na_2_SO_4_) and evaporated to dryness. The crude product was purified by column chromatography on silica gel with *n*-hexane/EtOAc 19:1.

*5-((1R,5S)-6,6-dimethylbicyclo[3.1.1]hept-2-en-2-yl)-3-phenyl-1,2,4-oxadiazole* (**6**): Yield: 73%, colorless oil. [α]${}_{D}^{20}$ = −11 (c 0.250, MeOH). ^1^H NMR (400 MHz, DMSO): δ 8.03–7.98 (2H, m), 7.62–7.52 (3H, m), 7.05–7.00 (1H, m), 2.99 (1H, dt, *J* = 1.3 Hz, 5.8 Hz), 2.66–2.52 (3H, m), 2.24–2.18 (1H, m), 1.39 (3H, s), 1.23 (1H, d, *J* = 9.1 Hz), 0.83 (3H, s). ^13^C NMR (100 MHz, DMSO): δ 174.4, 167.8, 135.5, 132.4, 131.4, 129.1, 127.0, 126.2, 42.0, 39.6, 37.4, 32.1, 30.7, 25.6, 25.5, 20.7 (Figure S4). Anal. calcd. for C_17_H_10_N_2_O: C, 76.66; H, 6.81; N, 10.52. Found: C, 76.70; H, 6.75; N, 10.44.

*3-(4-Chlorophenyl)-5-((1R,5S)-6,6-dimethylbicyclo[3.1.1]hept-2-en-2-yl)-1,2,4-oxadiazole* (**7**): Yield: 81%, colorless oil. [α]${}_{D}^{20}$ = −11 (c 0.250, MeOH). ^1^H NMR (400 MHz, DMSO): δ 8.02 (2H, d, *J* = 8.6 Hz), 7.63 (2H, d, *J* = 8.6 Hz), 7.05–7.02 (1H, m), 2.98 (1H, dt, *J* = 1.1 Hz, 5.6 Hz), 2.66–2.53 (3H, m), 2.24–2.18 (1H, m), 1.38 (3H, s), 1.23 (1H, d, *J* = 9.2 Hz), 0.83 (3H, s). ^13^C NMR (100 MHz, DMSO): δ 174.8, 167.0, 136.2, 135.9, 132.4, 129.3, 128.8, 125.1, 42.0, 39.6, 37.4, 32.1, 30.6, 25.6, 20.7 (Figure S5). Anal. calcd. for C_17_H_17_ClN_2_O: C, 67.88; H, 5.70; N, 9.31. Found: C, 67.90; H, 5.74; N, 9.24.

*5-((1R,6S)-7,7-dimethylbicyclo[4.1.0]hept-2-en-3-yl)-3-phenyl-1,2,4-oxadiazole* (**8**): Yield: 89%, white crystals, m.p.: 50–52 °C. [α]${}_{D}^{20}$ = +190 (c 0.250, MeOH). ^1^H NMR (400 MHz, CDCl_3_): δ 8.12–8.05 (2H, m), 7.50–7.40 (4H, m), 2.73–2.65 (1H, m), 2.31–2.21 (1H, m), 2.06–1.88 (1H, m), 1.42–1.36 (1H, m), 1.26 (1H, dt, *J* = 2.4 Hz, 8.2 Hz), 1.21 (3H, s), 0.99 (3H, s). ^13^C NMR (100 MHz, DMSO): δ 176.0, 167.7, 138.4, 131.3, 129.1, 126.9, 126.4, 120.4, 29.5, 28.7, 25.1, 23.5, 21.7, 16.2, 15.7 (Figure S6). Anal. calcd. for C_17_H_18_N_2_O: C, 76.66; H, 6.81; N, 10.52. Found: C, 76.81; H, 6.69; N, 10.60.

### 4.4. General Procedure for the Preparation of ***12*** and ***15***

**1** or **2** (6.02 mmol) and CDI (9.07 mmol) were dissolved in dry CH_2_Cl_2_ (75 mL). The mixture was stirred at room temperature for 1 h, then the solvent was evaporated and the residue was dissolved in anhydrous DMF (60 mL). After adding benzhydrazide (12.05 mmol), the mixture was stirred for 40 h at room temperature then evaporated to dryness. The residue was dissolved in water (50 mL) and the aqueous phase was extracted with CH_2_Cl_2_ (3 × 50 mL). The organic phase was dried (Na_2_SO_4_) and concentrated in vacuo.

*(1R,5S)-N′-benzoyl-6,6-dimethylbicyclo[3.1.1]hept-2-ene-2-carbohydrazide* (**12**): The crude product was purified by column chromatography on silica gel with *n*-hexane/EtOAc 1:1. Yield: 42%, white crystals, m.p.: 178–181 °C. [α]${}_{D}^{20}$ = −21 (c 0.250, MeOH). ^1^H NMR (400 MHz, DMSO): δ 10.21 (1H, s), 9.83 (1H, s), 7.88 (2H, d, *J* = 7.4 Hz), 7.61–7.44 (3H, m), 6.56 (1H, s), 2.72 (1H, t, *J* = 5.3 Hz), 2.49–2.29 (3H, m), 2.15–2.08 (1H, m), 1.31 (3H, s), 1.07 (1H, d, *J* = 8.8 Hz), 0.79 (3H, s). ^13^C NMR (100 MHz, DMSO): δ 165.7, 165.6, 141.4, 132.7, 131.6, 129.8, 128.8, 128.4, 127.3, 127.0, 40.9, 39.9, 37.2, 31.4, 30.8, 25.8, 20.8 (Figure S11). Anal. calcd. for C_17_H_20_N_2_O_2_: C, 71.81; H, 7.09; N, 9.85. Found: C, 71.89; H, 7.08; N, 9.85.

*(1R,6S)-N′-benzoyl-7,7-dimethylbicyclo[4.1.0]hept-2-ene-3-carbohydrazide* (**15**): The crude product was purified by column chromatography on silica gel with *n*-hexane/EtOAc 3:1. Yield: 55%, white crystals, m.p.: 188–191 °C. [α]${}_{D}^{20}$> = +79 (c 0.250, MeOH). ^1^H NMR (400 MHz, DMSO) δ 10.21 (1H, br s), 9.75 (1H, br s), 7.89 (2H, d, *J* = 7.66 Hz), 7.62–7.48 (3H, m), 6.92 (1H, d, *J* = 4.93 Hz), 2.38–2.27 (1H, m), 1.96–1.72 (3H, m), 1.32–1.24 (1H, m), 1.16 (3H, s), 1.19–1.11 (1H, m), 0.92 (3H, s). ^13^C NMR (100 MHz, DMSO): δ 132.8, 132.7, 131.6, 129.5, 128.4, 127.3, 28.6, 27.2, 24.0, 22.7, 21.5, 16.7, 15.6 (Figure S14). Anal. calcd. for C_17_H_20_N_2_O_2_: C, 71.81; H, 7.09; N, 9.85. Found: C, 71.84; H, 7.11; N, 9.80.

### 4.5. General Procedure for the Preparation of ***13*** and ***16***

**12** or **15** (3.52 mmol) was heated in POCl_3_ (28 mL) at 80 °C for 3 h then the reaction mixture was cooled to room temperature, poured onto ice and made basic (pH 8) with saturated NaHCO_3_ solution. The aqueous phase was extracted with CH_2_Cl_2_ (3 × 100 mL), then the organic phase was dried (Na_2_SO_4_) and evaporated to dryness. The crude product was purified by column chromatography on silica gel with *n*-hexane/EtOAc 4:1.

*2-((1R,5S)-6,6-dimethylbicyclo[3.1.1]hept-2-en-2-yl)-5-phenyl-1,3,4-oxadiazole* (**13**): Yield: 38%, white crystals, m.p.: 54–56 °C. [α]${}_{D}^{20}$ = −52 (c 0.250, MeOH). ^1^H NMR (400 MHz, DMSO): δ 8.09–8.04 (2H, m), 7.53–7.46 (3H, m), 6.74–6.70 (1H, m), 3.14 (1H, dt, *J* = 1.4 Hz, 5.9 Hz), 2.63–2.48 (3H, m), 2.26–2.20 (1H, m), 1.41 (3H, s), 1.31 (1H, d, *J* = 9.3 Hz), 0.89 (3H, s). ^13^C NMR (100 MHz, DMSO): δ 163.4, 131.9, 131.8, 130.8, 129.3, 126.5, 123.3, 41.6, 39.8, 37.4, 31.8, 30.6, 25.6, 20.7 (Figure S12). Anal. calcd. for C_17_H_18_N_2_O: C, 76.66; H, 6.81; N, 10.52. Found: C, 76.81; H, 6.59; N, 10.59.

*2-Phenyl-5-(4-(propan-2-ylidene)cyclohex-1-en-1-yl)-1,3,4-oxadiazole* (**16**): Yield: 15%, yellow crystals, m.p.: 87–90 °C. ^1^H NMR (400 MHz, DMSO): δ 8.10–8.06 (2H, m), 7.64–7.57 (3H, m), 7.56 (1H, s), 2.56 (2H, t, *J* = 5.9 Hz), 2.38 (2H, t, *J* = 5.9 Hz), 1.96 (3H, s), 1.84 (3H, s), 1.80–1.73 (2H, m). ^13^C NMR (100 MHz, DMSO): δ 165.3, 163.1, 135.2, 131.7, 129.6, 129.3, 127.0, 126.5, 123.4, 119.6, 25.4, 24.0, 21.6, 21.1, 20.0 (Figure S15). Anal. calcd. for C_17_H_18_N_2_O: C, 76.66; H, 6.81; N, 10.52. Found: C, 76.54; H, 6.86; N, 10.55.

### 4.6. General Procedure for Dihydroxylation

To compounds **6**–**8**, **13** and **16** (1.09 mmol) in acetone (30 mL) NMO (0.99 mL, 50% aqueous solution) and OsO_4_ (0.32 mL, 2% *t*-BuOH solution) were added. The reaction mixture was stirred for 24 h at room temperature. The reaction was then quenched by the addition of saturated Na_2_SO_3_ solution (30 mL) and extracted with EtOAc (3 × 50 mL). The organic layer was dried (Na_2_SO_4_) and evaporated in *vacuo*.

*(1R,2R,3S,5R)-6,6-dimethyl-2-(3-phenyl-1,2,4-oxadiazol-5-yl)bicyclo[3.1.1]heptane-2,3-diol* (**9**): The crude product was purified by column chromatography on silica gel with *n*-hexane/EtOAc 4:1. Yield: 80%, white crystals, m.p.: 133–136 °C. [α]${}_{D}^{20}$ = −19 (c 0.250, MeOH). ^1^H NMR (400 MHz, DMSO) δ 8.05–7.99 (2H, m), 7.64–7.55 (3H, m), 5.96 (1H, d, *J* = 5.3 Hz), 5.62 (1H, s), 4.96–4.88 (1H, m), 2.69 (1H, t, *J* = 5.6 Hz), 2.49–2.42 (1H, m), 2.31–2.22 (1H, m), 1.96–1.89 (1H, m), 1.80 (1H, dt, *J* = 3.4, 14.1 Hz), 1.55 (1H, d, *J* = 10.5 Hz), 1.26 (3H, s), 0.56 (3H, s). ^13^C NMR (DMSO, 100 MHz) δ 183.62, 167.14, 131.5, 129.2, 127.0, 126.1, 72.3, 63.6, 50.0, 39.6, 37.9, 36.9, 26.8, 25.9, 21.9 (Figures S7 and S8). Anal. calcd. for C_17_H_20_N_2_O_3_: C, 67.98; H, 6.71; N, 9.33. Found: C, 67.88; H, 6.75; N, 9.41.

*(1R,2R,3S,5R)-2-(3-(4-chlorophenyl)-1,2,4-oxadiazol-5-yl)-6,6-dimethylbicyclo[3.1.1]heptane-2,3-diol* (**10**): The crude product was purified by column chromatography on silica gel with *n*-hexane/EtOAc 4:1. Yield: 95%, white crystals, m.p.: 114–117 °C. [α]${}_{D}^{20}$ = −20 (c 0.250, MeOH). ^1^H NMR (400 MHz, DMSO): δ 8.04 (2H, d, J = 8.5 Hz), 7.67 (2H, d, J = 8.5 Hz), 5.97 (1H, d, *J* = 6.5 Hz), 5.65 (1H, s), 5.96–4.89 (1H, m), 2.69 (1H, t, *J* = 5.8 Hz), 2.50–2.43 (1H, m), 2.30–2.23 (1H, m), 1.96–1.90 (1H, m), 1.81 (1H, dt, *J* = 14.0 Hz, 3.5 Hz), 1.56 (1H, d, *J* = 10.5 Hz), 1.26 (3H, s), 0.57 (3H, s). ^13^C NMR (100 MHz, DMSO): δ 183.9, 166.4, 136.3, 129.4, 128.8, 125.0, 72.4, 63.6, 50.0, 39.6, 37.8, 36.8, 26.8, 25.9, 21.9 (Figure S9). Anal. calcd. for C_17_H_19_ClN_2_O_3_: C, 60.99; H, 5.72; N, 8.37. Found: C, 61.09; H, 5.70; N, 8.40.

*(1R,2S,3R,6S)-7,7-dimethyl-3-(3-phenyl-1,2,4-oxadiazol-5-yl)bicyclo[4.1.0]heptane-2,3-diol* (**11**): The crude product was purified by column chromatography on silica gel with *n*-hexane/EtOAc 4:1. Yield: 41%, colorless oil. [α]${}_{D}^{20}$ = −6 (c 0.250, MeOH). ^1^H NMR (400 MHz, DMSO): δ 8.02–7.98 (2H, m), 7.61–7.53 (3H, m), 5.50 (1H, s), 5.15 (1H, d, *J* = 7.3 Hz), 3.60 (1H, dd, *J* = 3.1 Hz, 7.3 Hz), 2.02–1.81 (2H, m), 1.62–1.51 (2H, m), 1.07 (3H, s), 1.03 (3H, s), 0.80–0.73 (1H, m), 0.69–0.64 (1H, m). ^13^C NMR (100 MHz, DMSO): δ 185.0, 167.4, 131.3, 129.1, 127.0, 126.4, 73.2, 69.9, 32.8, 28.6, 25.9, 19.8, 16.1, 15.0, 14.3 (Figure S10). Anal. calcd. for C_17_H_20_N_2_O_3_: C, 67.98; H, 6.71; N, 9.33. Found: C, 67.88; H, 6.77; N, 9.38.

*(1R,2R,3S,5R)-6,6-dimethyl-2-(5-phenyl-1,3,4-oxadiazol-2-yl)bicyclo[3.1.1]heptane-2,3-diol* (**14**): The crude product was purified by column chromatography on silica gel with *n*-hexane/EtOAc 2:1. Yield: 95%, white crystals, m.p.: 110–112 °C. [α]${}_{D}^{20}$ = +11 (c 0.250, MeOH). ^1^H NMR (400 MHz, DMSO): δ 8.06–8.01 (2H, m), 7.68–7.59 (3H, m), 5.94 (1H, d, *J* = 6.3 Hz), 5.51 (1H, s), 4.94–4.88 (1H, m), 2.68 (1H, t, *J* = 5.7 Hz), 2.42–2.29 (1H, m), 2.29–2.21 (1H, m), 1.95–1.89 (1H, m), 1.78 (1H, dt, *J* = 14.0 Hz, 3.6 Hz), 1.55 (1H, d, *J* = 10.4 Hz), 1.25 (3H, s), 0.59 (3H, s). ^13^C NMR (100 MHz, DMSO): δ 170.4, 163.9, 132.0, 129.4, 126.5, 123.3, 71.7, 63.1, 49.7, 39.7, 37.9, 36.9, 27.0, 26.1, 22.0 (Figure S13). Anal. calcd. for C_17_H_20_N_2_O_3_: C, 67.98; H, 6.71; N, 9.33. Found: C, 68.05; H, 6.65; N, 9.30.

*1-(2-Hydroxypropan-2-yl)-4-(5-phenyl-1,3,4-oxadiazol-2-yl)cyclohex-3-enol* (**17**): The crude product was purified by column chromatography on silica gel with *n*-hexane/EtOAc 1:1. Yield: 55%, colorless oil. ^1^H NMR (400 MHz, DMSO): δ 8.07–8.02 (2H, m), 7.65–7.58 (3H, m), 6.99 (1H, s), 4.67 (1H, s), 4.30 (1H, s), 2.65–2.57 (1H, m), 2.31–2.20 (1H, m), 1.85–1.75 (2H, m), 1.71–1.62 (2H, m), 1.89 (3H, s), 1.09 (3H, s). ^13^C NMR (100 MHz, DMSO): δ 164.7, 163.3, 136.3, 131.9, 129.4, 126.5, 124.0, 123.3, 73.4, 72.4, 29.9, 25.0, 24.3, 24.2, 18.0 (Figure S16). Anal. calcd. for C_17_H_20_N_2_O_3_: C, 67.98; H, 6.71; N, 9.33. Found: C, 67.99; H, 6.59; N, 9.53.

### 4.7. Antiproliferative Assay

The human gynecological cancer cell lines isolated from cervical adenocarcinoma (HeLa), ovarian cancer (A2780), and breast cancers (MDA-MB-231 and MCF7) were purchased from European Collection of Cell Cultures (Salisbury, UK). The cells were grown in Minimum Essential Medium (MEM) supplemented with 10% fetal calf serum (FCS), 1% non-essential amino acids, and 1% penicillin-streptomycin. All media and supplements for these experiments were obtained from Lonza Group Ltd. (Basel, Switzerland). The cells were maintained at 37 °C in humidified atmosphere containing 5% CO_2_. The antiproliferative properties of the prepared monoterpene-based oxadiazoles were determined by the MTT [3-(4,5-dimethylthiazol-2-yl)-2,5-diphenyltetrazolium bromide] assay against adherent cancer cell lines [41]. Briefly, cells were seeded into 96 well plates (5000 cells/well) and incubated with two concentrations of the tested compounds (10 and 30 μM) under cell-culturing conditions. After incubation for 72 h, MTT solution (5 mg/mL) was added to each sample which were incubated for further 4 h. The formazan crystals precipitated were dissolved in 100 μL dimethyl sulfoxide, and the absorbance was measured at 545 nm with a microplate reader (Awareness Technology, Palm City, FL, USA). Two independent experiments were performed with five wells for each condition. Cisplatin (Ebewe GmbH, Unterach, Austria), a clinically used anticancer agent, was used as a reference agent. It the case of the most effective agents, the assay was repeated with a set of concentrations (0.01–30 μM) in order to determine the IC_50_ values. Calculations were done by means of the GraphPad Prism 5.01 software (GraphPad Software Inc., San Diego, CA, USA) using the non-linear regression model log (inhibitor) vs. normalized response and variable slope fit.

## Figures and Tables

**Table 1:** Influence of catalyst on the reaction yield and enantioselectivity according to Scheme 4.

Entry	Catalyst	Yield (%) ^a^	*ee* (%) ^b^	Configuration of the Major Enantiomer ^c^
1	9	87	74	*S*
2	10	85	62	*S*
3	11	86	50	*S*
4	14	83	70	*S*

^a^ Yields obtained after chromatography on silica column; ^b^ Determined with the crude product by GC (Chirasil-DEX CB column); ^c^ Determined by comparing the *t_R_* of GC analysis and the optical rotation with literature data [39,40].

**Table 2:** Antiproliferative activities of the tested monoterpene analogs.

Analog	Conc. (µM)	Inhibition (%) ± SEM [Calculated IC_50_ Value (µM)]			
		HeLa	A2780	MCF7	MDA-MB-231
3	10	29.83 ± 1.65	96.66 ± 0.23	35.07 ± 2.64	19.06 ± 2.98
	30	98.46 ± 0.08	96.60 ± 0.37	93.00 ± 0.68	84.58 ± 1.87
		[12.23]	[1.44]	[12.37]	[16.47]
4	10	37.96 ± 2.20	96.28 ± 0.32	49.99 ± 0.80	33.33 ± 1.94
	30	98.17 ± 0.19	96.82 ± 0.18	96.08 ± 0.52	89.95 ± 0.90
		[11.46]	[1.91]	[10.02]	[13.02]
5	10	21.94 ± 2.94	91.95 ± 0.26	28.62 ± 1.98	20.31 ± 1.45
	30	96.31 ± 0.33	93.77 ± 0.27	85.27 ± 1.84	58.06 ± 1.75
		[13.62]	[2.05]	[14.54]	[24.28]
6	10	– *	56.80 ± 3.18	17.30 ± 1.74	19.61 ± 2.23
	30	47.39 ± 2.99	93.34 ± 0.69	23.87 ± 2.37	29.68 ± 2.54
7	10	–	15.35 ± 1.79	19.80 ± 1.99	–
	30	24.18 ± 2.42	36.92 ± 1.39	23.44 ± 1.86	24.66 ± 1.38
8	10	–	12.18 ± 1.70	14.62 ± 2.49	11.71 ± 0.74
	30	12.31 ± 2.25	36.58 ± 1.38	35.75 ± 2.79	20.27 ± 2.62
9	10	–	–	–	11.45 ± 2.75
	30	10.83 ± 2.40	34.56 ± 1.83	–	21.57 ± 1.69
10	10	–	17.93 ± 1.06	–	11.43 ± 1.68
	30	26.01 ± 0.74	48.27 ± 0.64	44.00 ± 1.34	24.67 ± 2.13
11	10	12.36 ± 1.23	–	–	–
	30	13.41 ± 1.87	28.44 ± 0.92	25.32 ± 1.10	10.36 ± 2.14
12	10	–	–	–	15.51 ± 2.77
	30	–	31.13 ± 2.48	–	15.92 ± 2.69
13	10	–	11.76 ± 0.76	–	–
	30	–	48.66 ± 1.97	–	18.81 ± 2.59
14	10	–	–	–	–
	30	–	29.12 ± 2.32	14.46 ± 2.31	16.28 ± 1.85
cisplatin	10	42.61 ± 2.33	83.57 ± 1.21	53.03 ± 2.29	67.51 ± 1.01
	30	99.93 ± 0.26	95.02 ± 0.28	86.90 ± 1.24	87.75 ± 1.10
		[12.43]	[1.30]	[5.78]	[3.74]

* Growth inhibition values less than 10% are considered negligible and not given numerically.

## Supplementary Materials

Supplementary materials can be found at www.mdpi.com/1422-0067/19/1/81/s1.

## Abbreviations

DCM	dichloromethane
CDI	1,1′-carbonyldiimidazol
TBAF	tetrabutylammonium fluoride
NMO	4-methylmorpholine *N*-oxide
