# Supplementary material for: Stereoselective Synthesis, Synthetic and Pharmacological Application of Monoterpene-Based 1,2,4- and 1,3,4-Oxadiazoles
Source: Int J Mol Sci. 2017 Dec 28;19(1):81. doi: 10.3390/ijms19010081 (PMC5796031; doi:10.3390/ijms19010081)
Supplement: Supplementary file 1 [file ijms-19-00081-s001.pdf]

Supplementary materials: Stereoselective synthesis, synthetic and pharmacological application of monoterpene-based 1,2,4- and 1,3,4-oxadiazols

Tímea Gonda, Péter Bérdi, István Zupkó, Ferenc Fülöp and Zsolt Szakonyi

Contents

^{1}H and ^{13}C NMR spectra of new compounds S1-S16

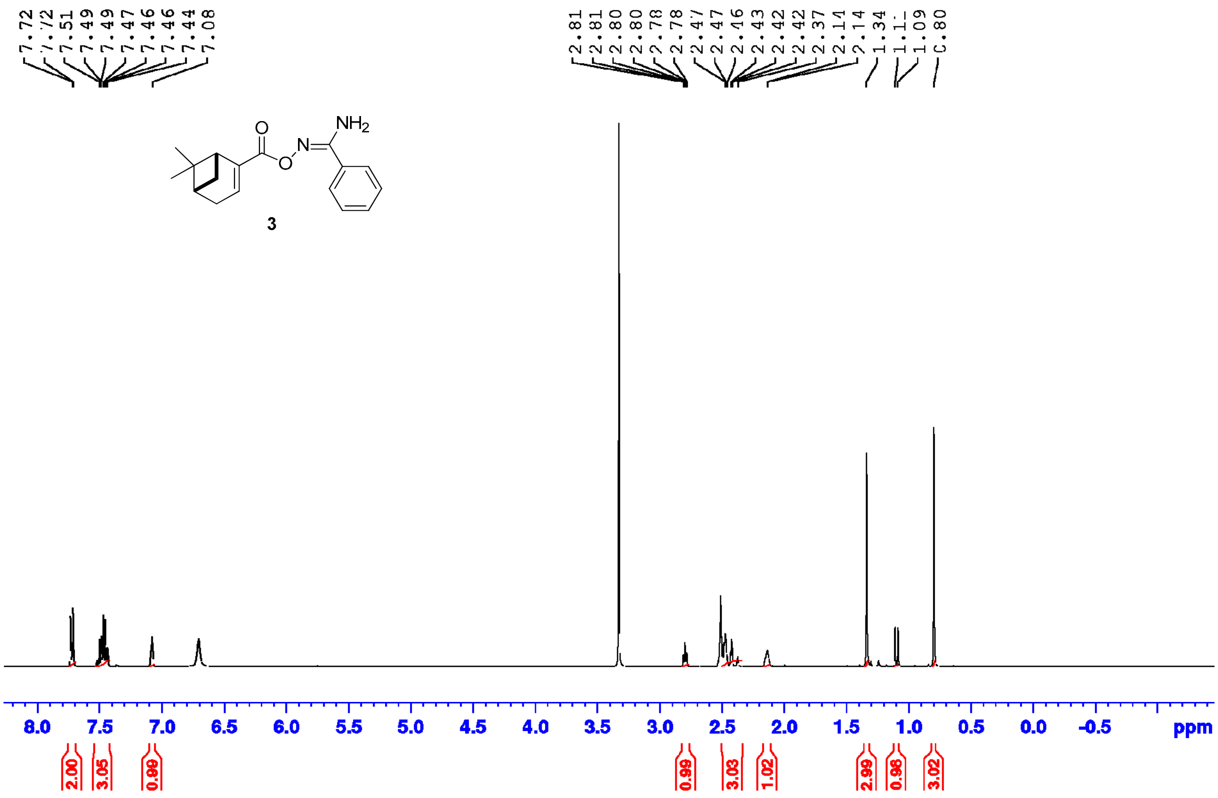

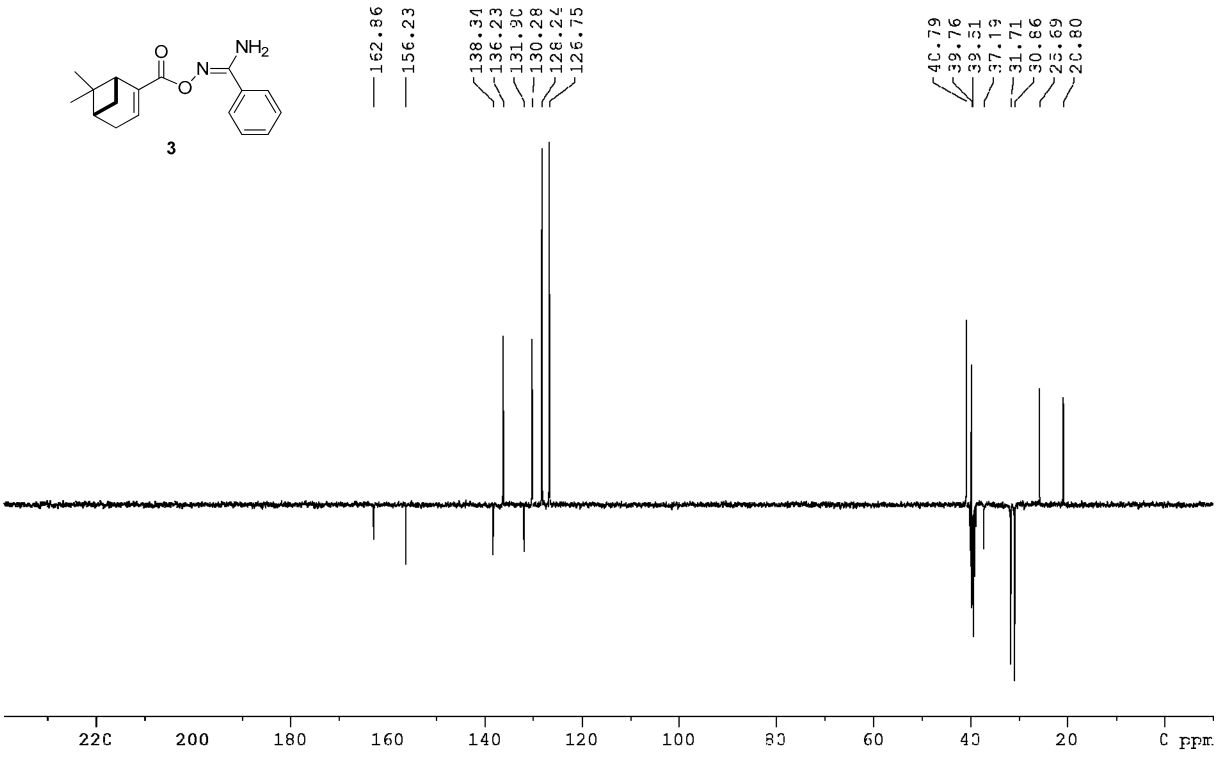

^{1}H- and ^{13}C-NMR spectrum of *(E)-N'-(((1R,5S)-6,6-dimethylbicyclo[3.1.1]hept-2-ene-2-carbonyl)oxy)benzimidamide* (**3**)

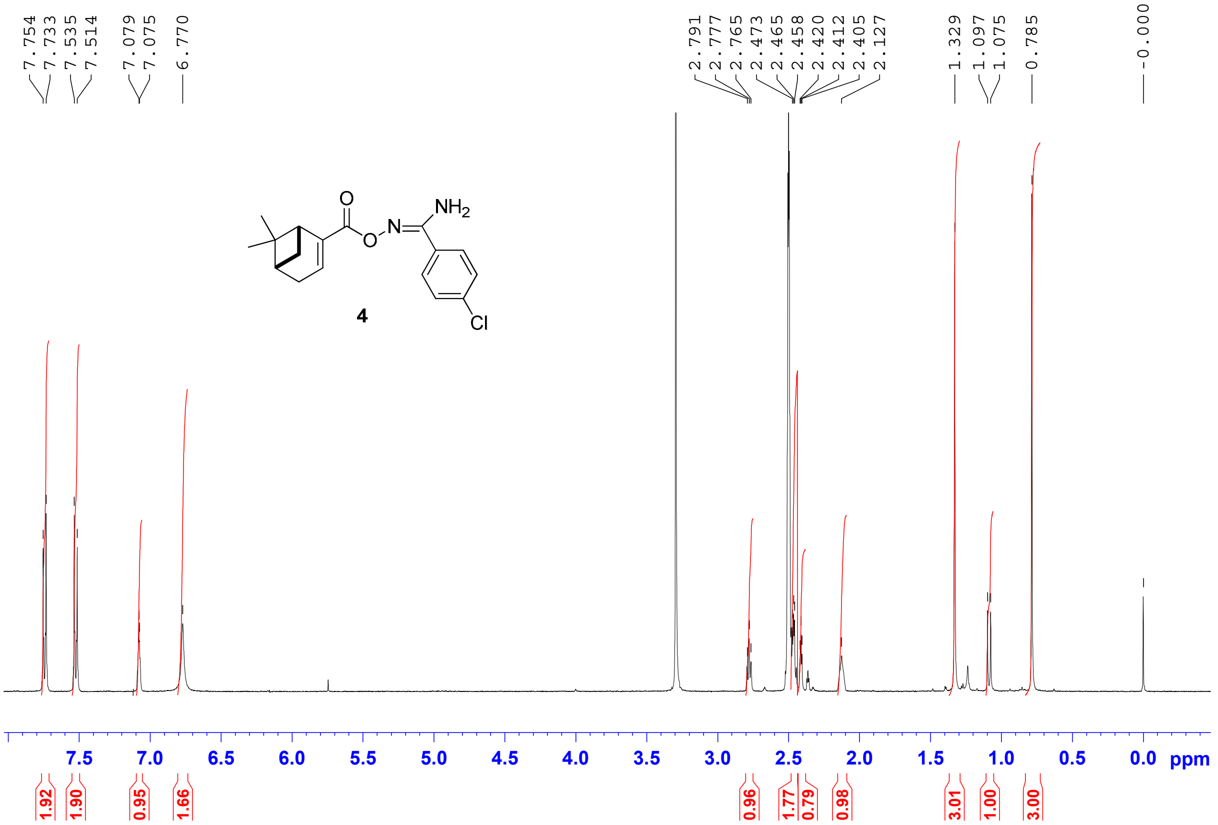

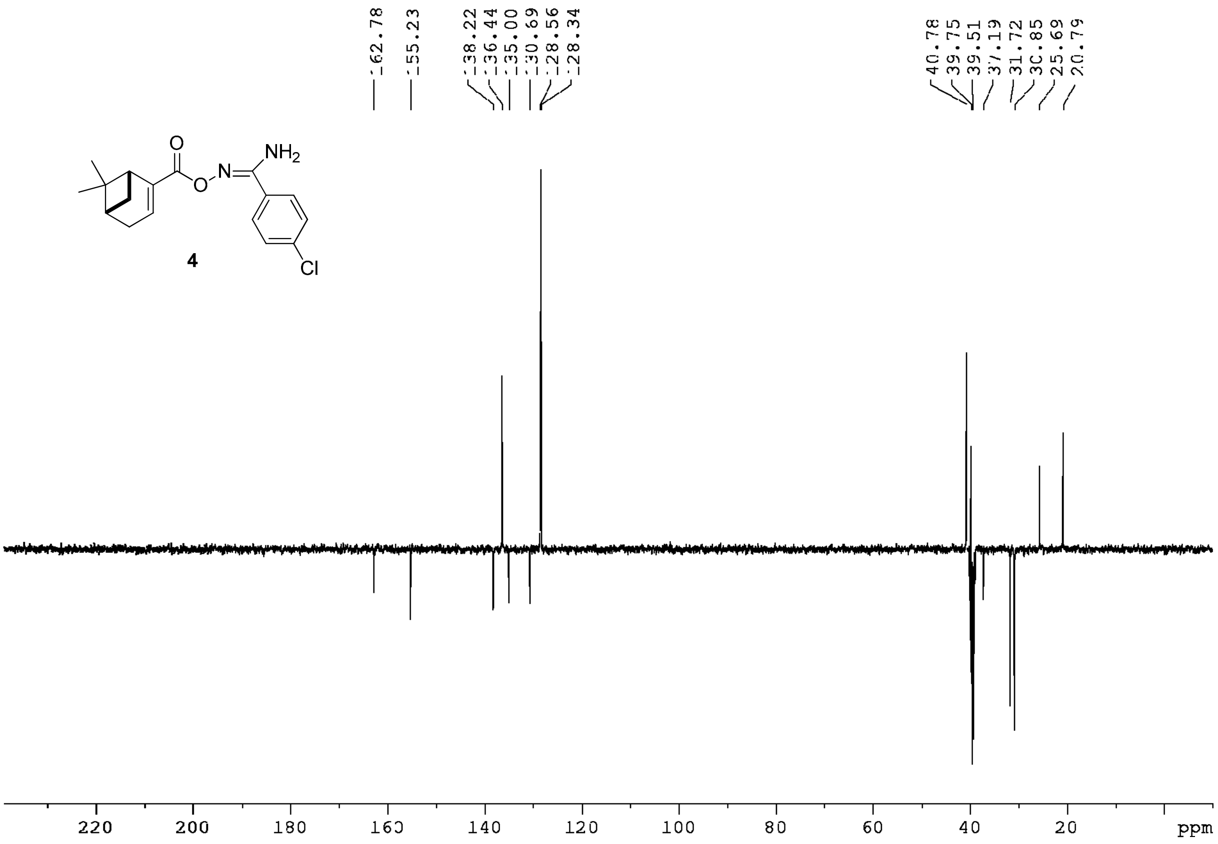

^{1}H- and ^{13}C-NMR spectrum of *(E)-4-chloro-N'-(((1R,5S)-6,6-dimethylbicyclo[3.1.1]hept-2-ene-2-carbonyl)oxy)benzimidamide* (**4**)

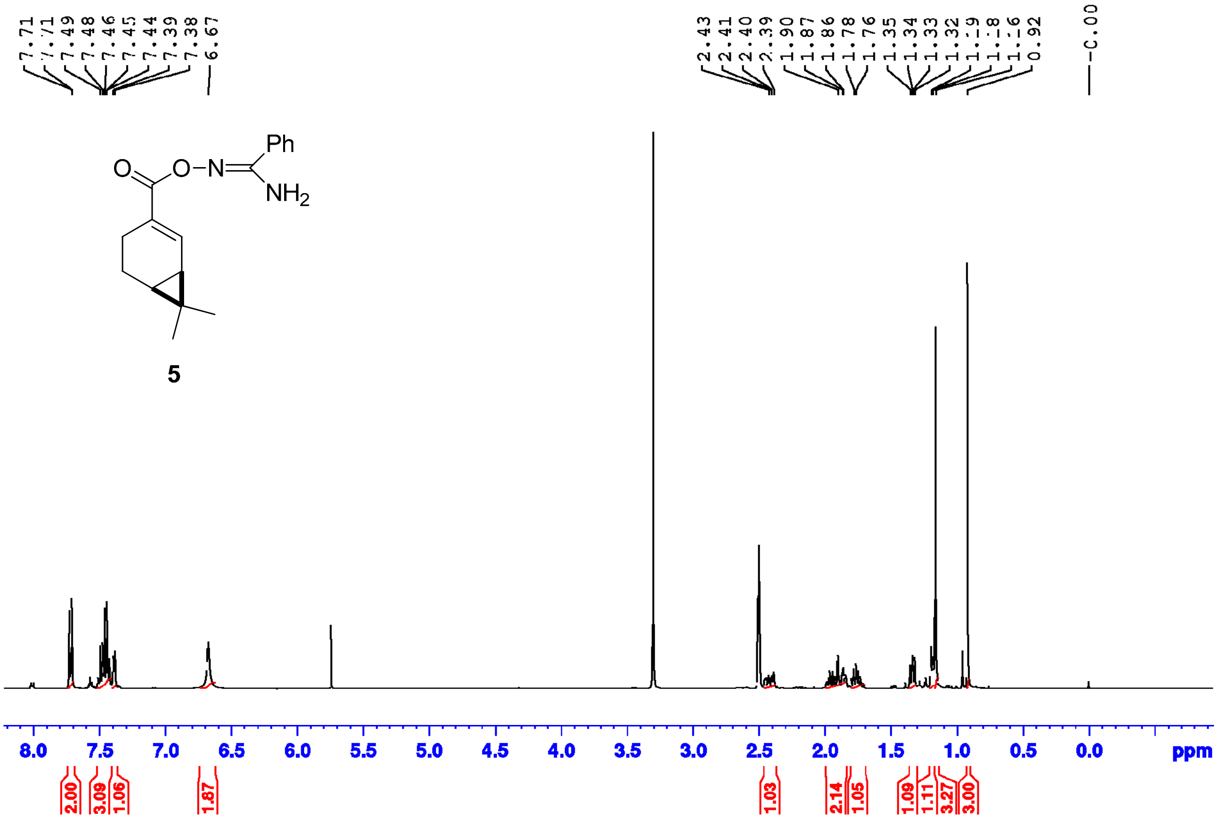

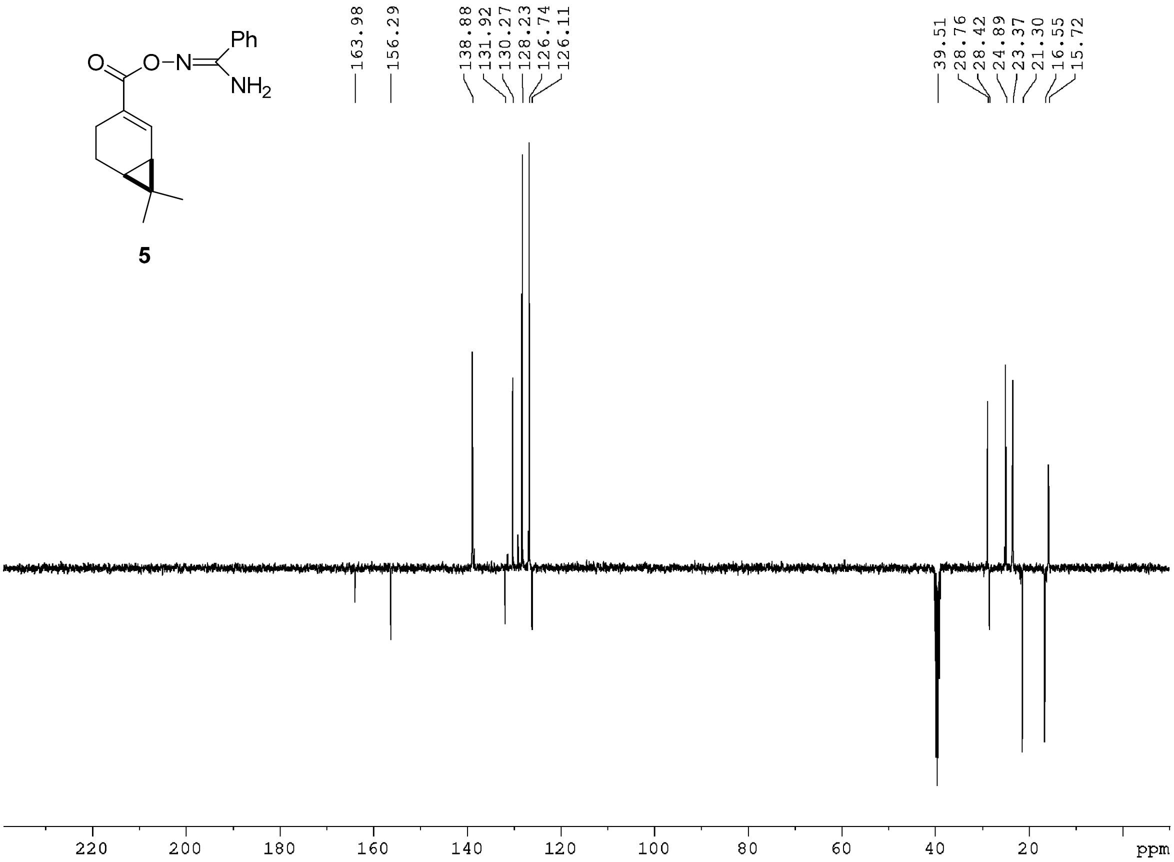

^{1}H- and ^{13}C-NMR spectrum of *N'-(((1R,6S)-7,7-dimethylbicyclo[4.1.0]hept-2-ene-3-carbonyl)oxy)benzimidamide* (**5**)

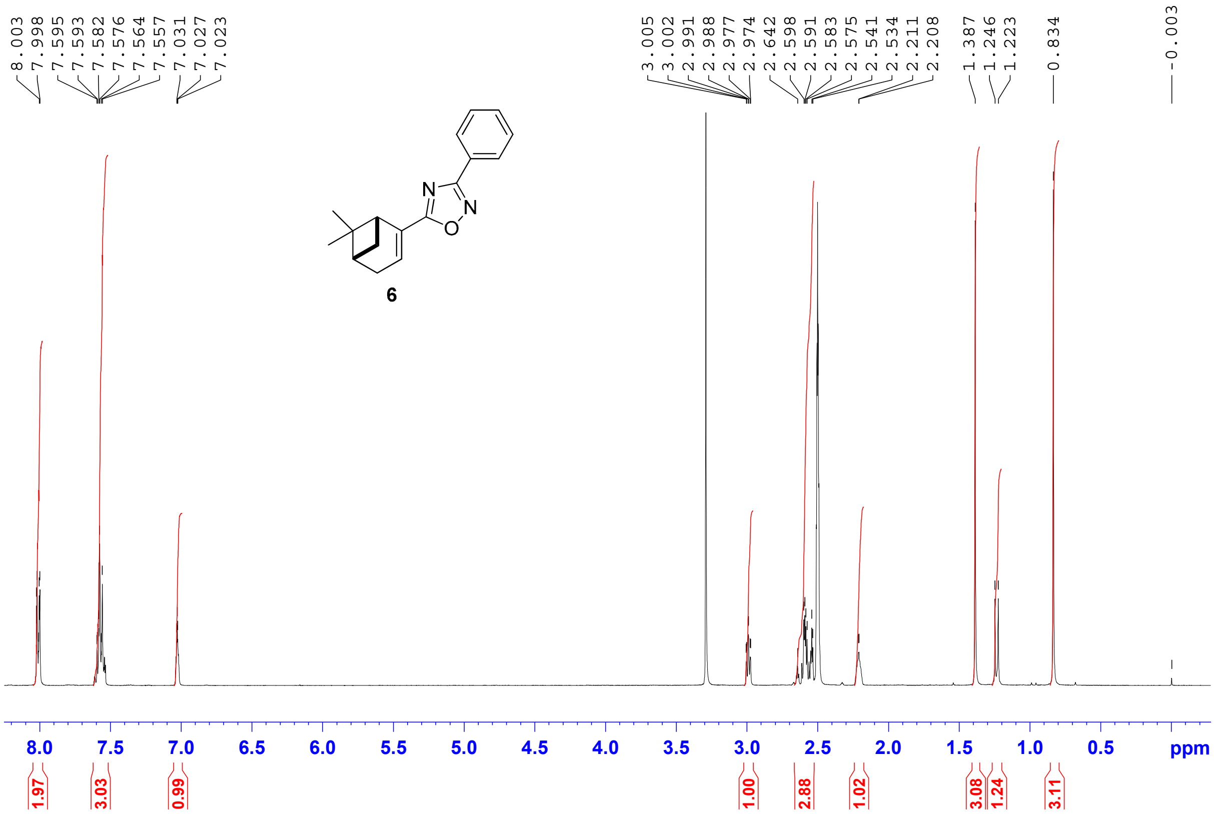

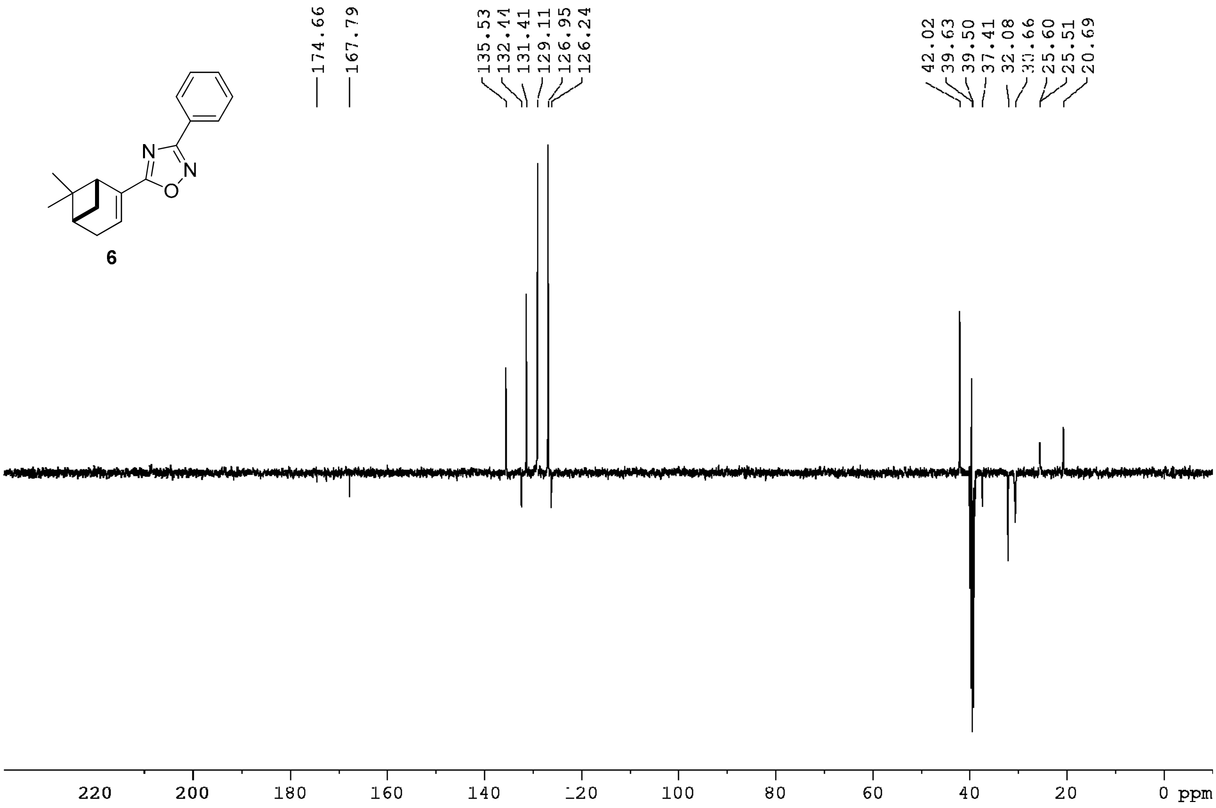

^{1}H- and ^{13}C-NMR spectrum of *5-((1R,5S)-6,6-dimethylbicyclo[3.1.1]hept-2-en-2-yl)-3-phenyl-1,2,4-oxadiazole* (**6**)

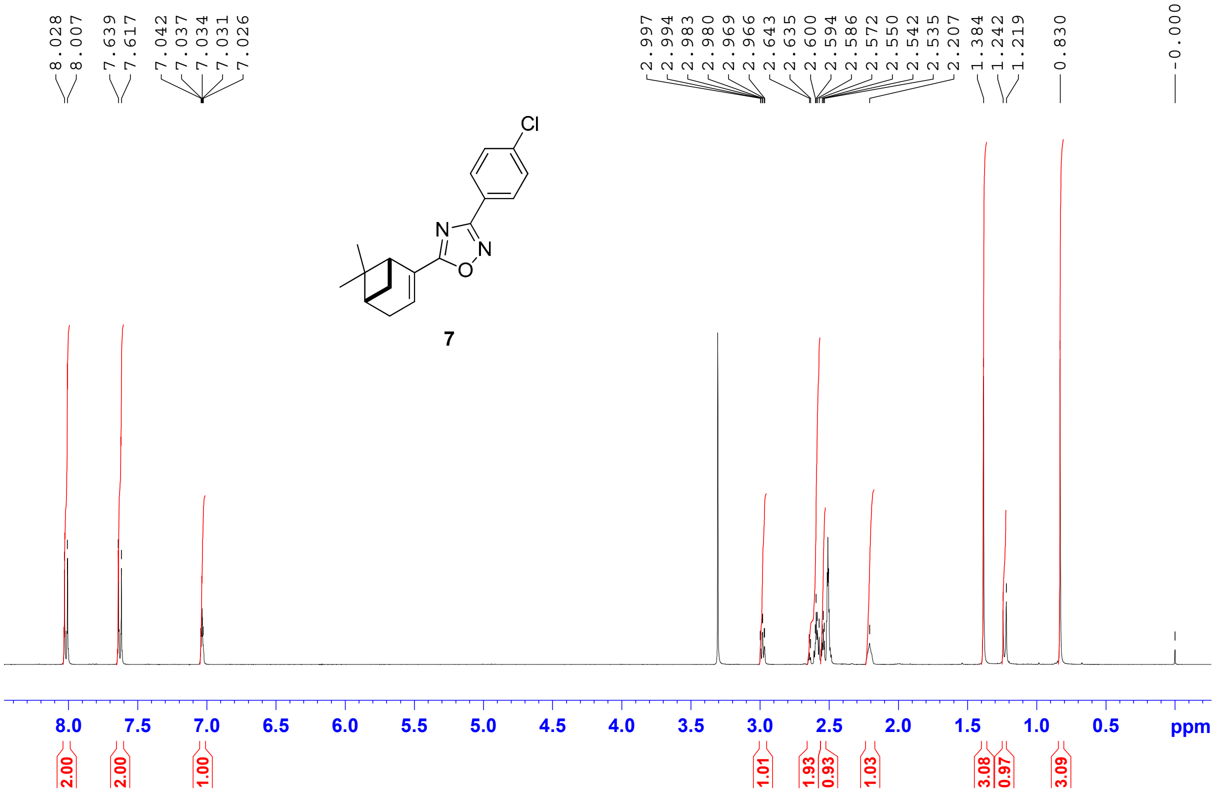

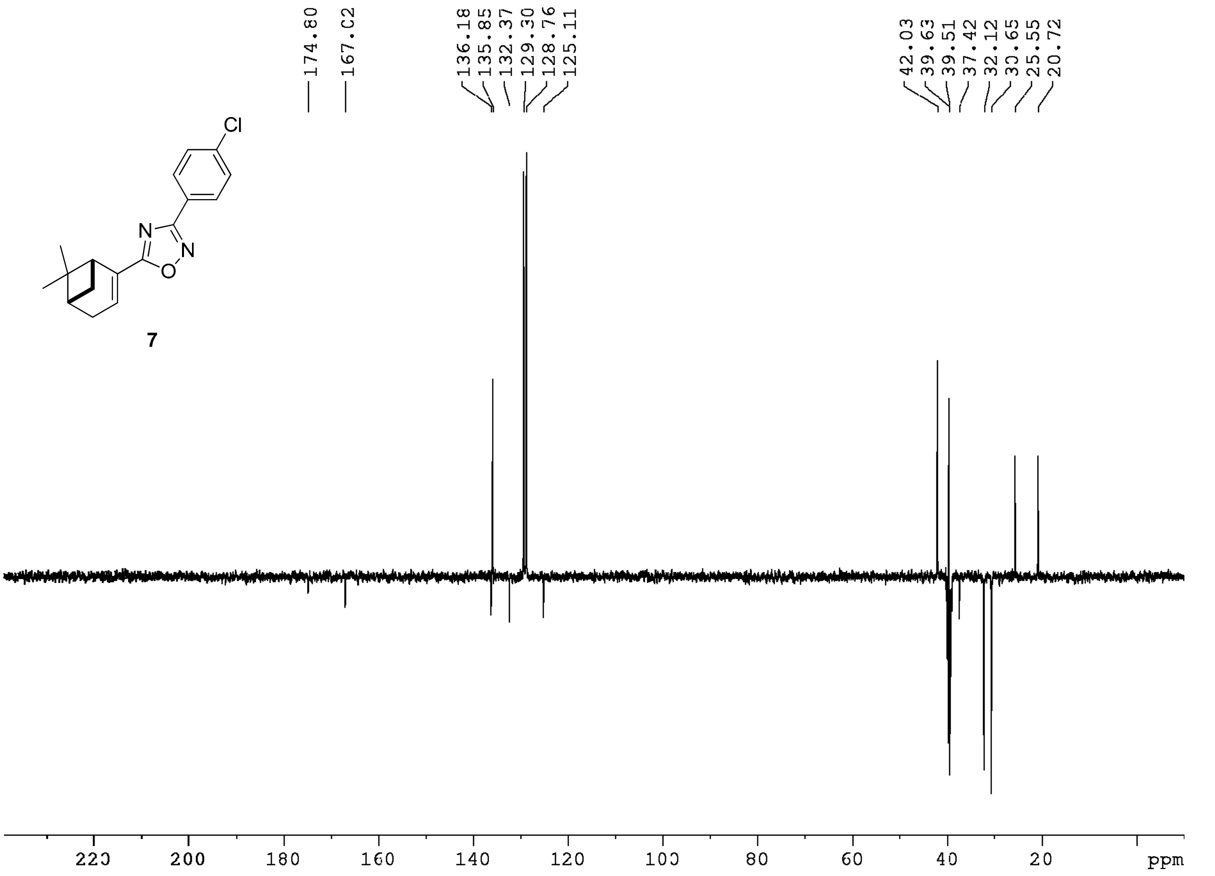

^{1}H- and ^{13}C-NMR spectrum of *3-(4-chlorophenyl)-5-((1R,5S)-6,6-dimethylbicyclo[3.1.1]hept-2-en-2-yl)-1,2,4-oxadiazole* (**7**)

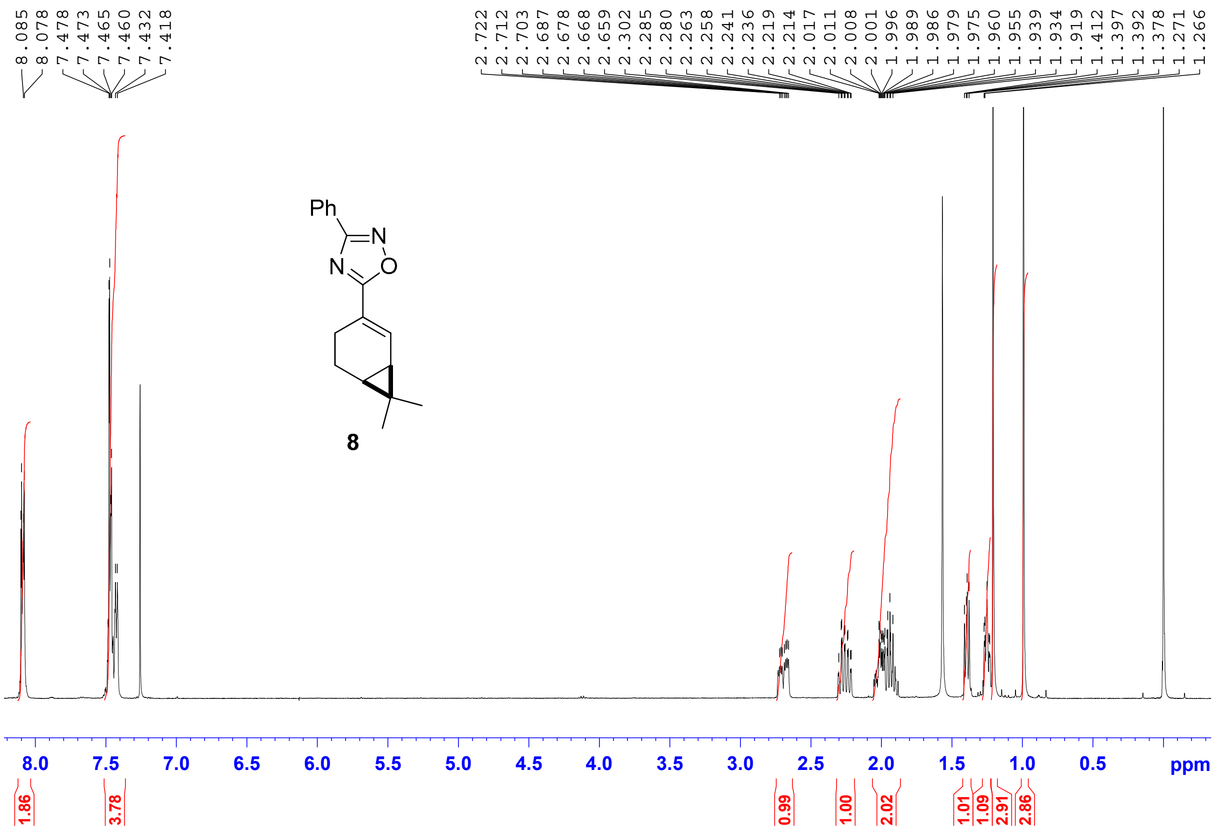

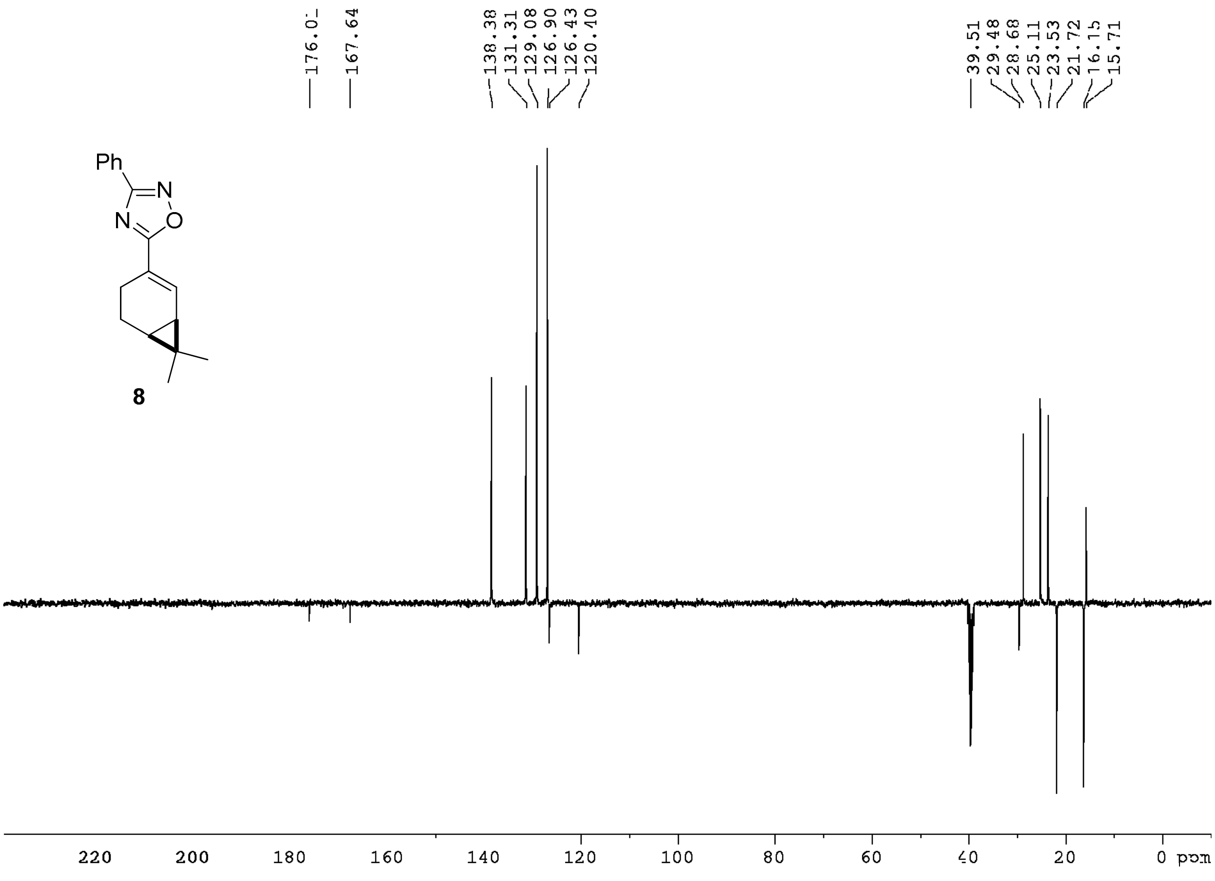

^{1}H- and ^{13}C-NMR spectrum of *5-((1R,6S)-7,7-dimethylbicyclo[4.1.0]hept-2-en-3-yl)-3-phenyl-1,2,4-oxadiazole* (**8**)

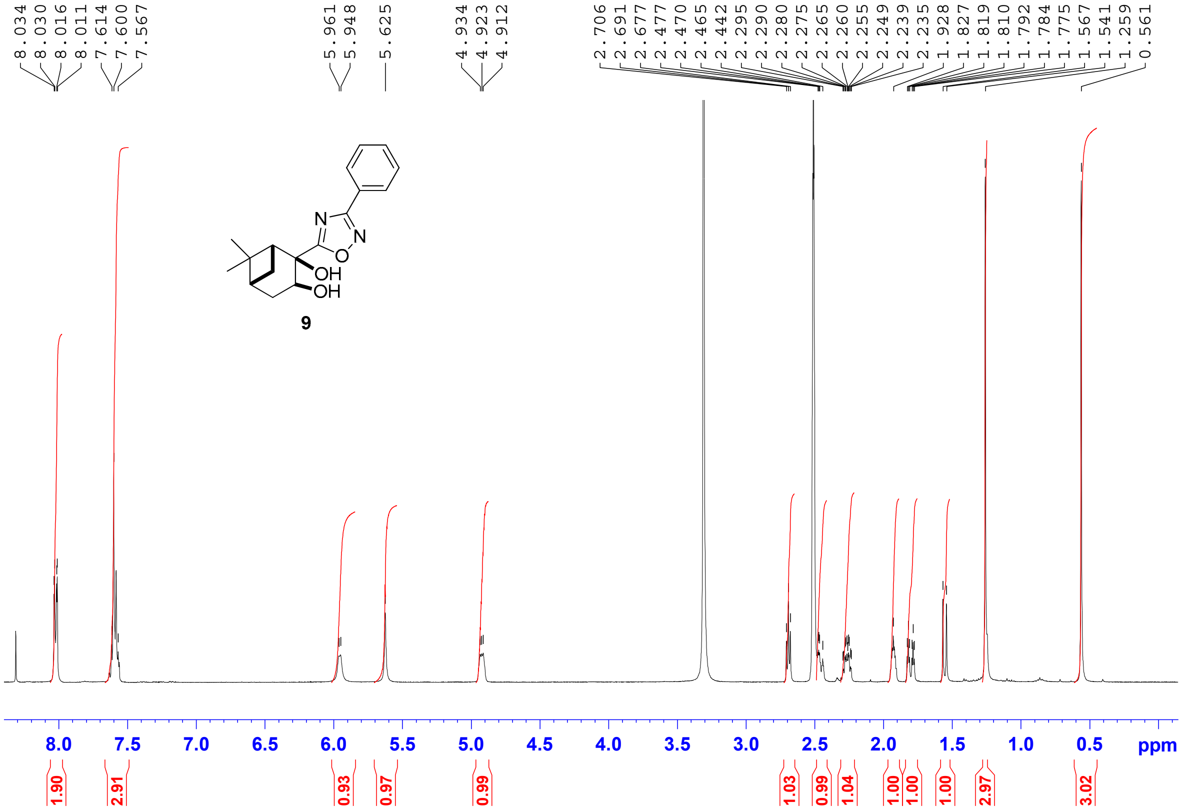

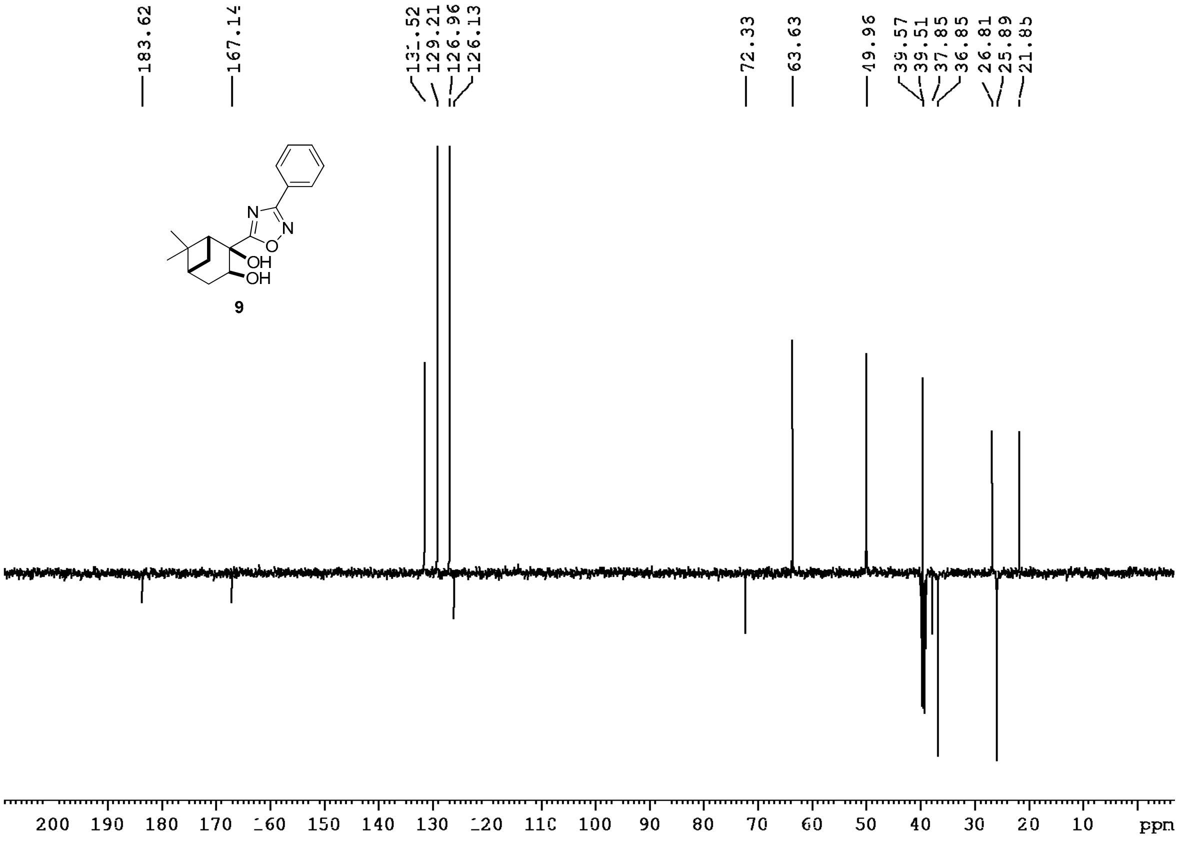

^{1}H- and ^{13}C-NMR spectrum of *(1R,2R,3S,5R)-6,6-dimethyl-2-(3-phenyl-1,2,4-oxadiazol-5-yl)bicyclo[3.1.1]heptane-2,3-diol* (**9**)

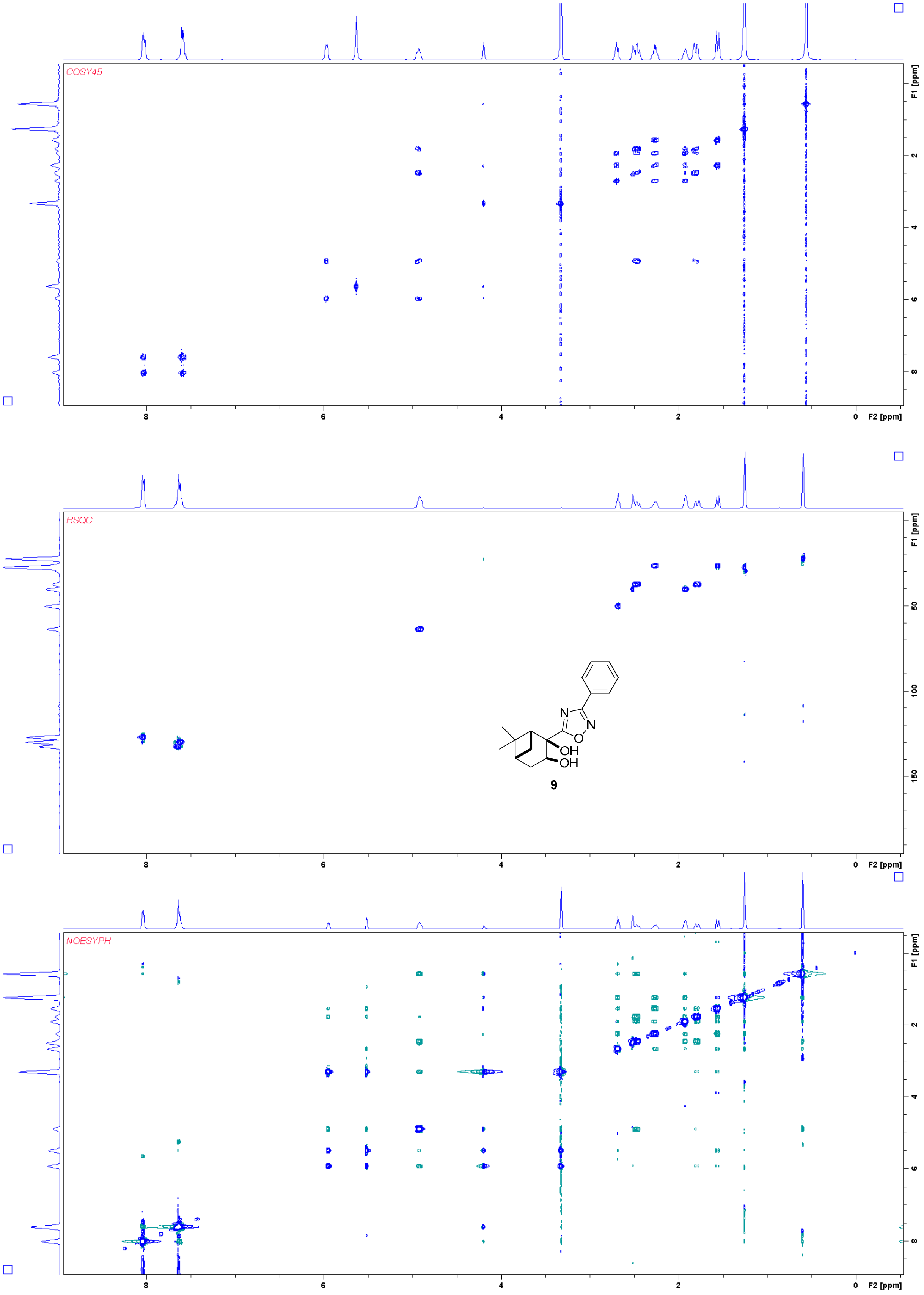

COSY-, HSQC and NOESY spectrum of *(1R,2R,3S,5R)-6,6-dimethyl-2-(3-phenyl-1,2,4-oxadiazol-5-yl)bicyclo[3.1.1]heptane-2,3-diol* (**9**)

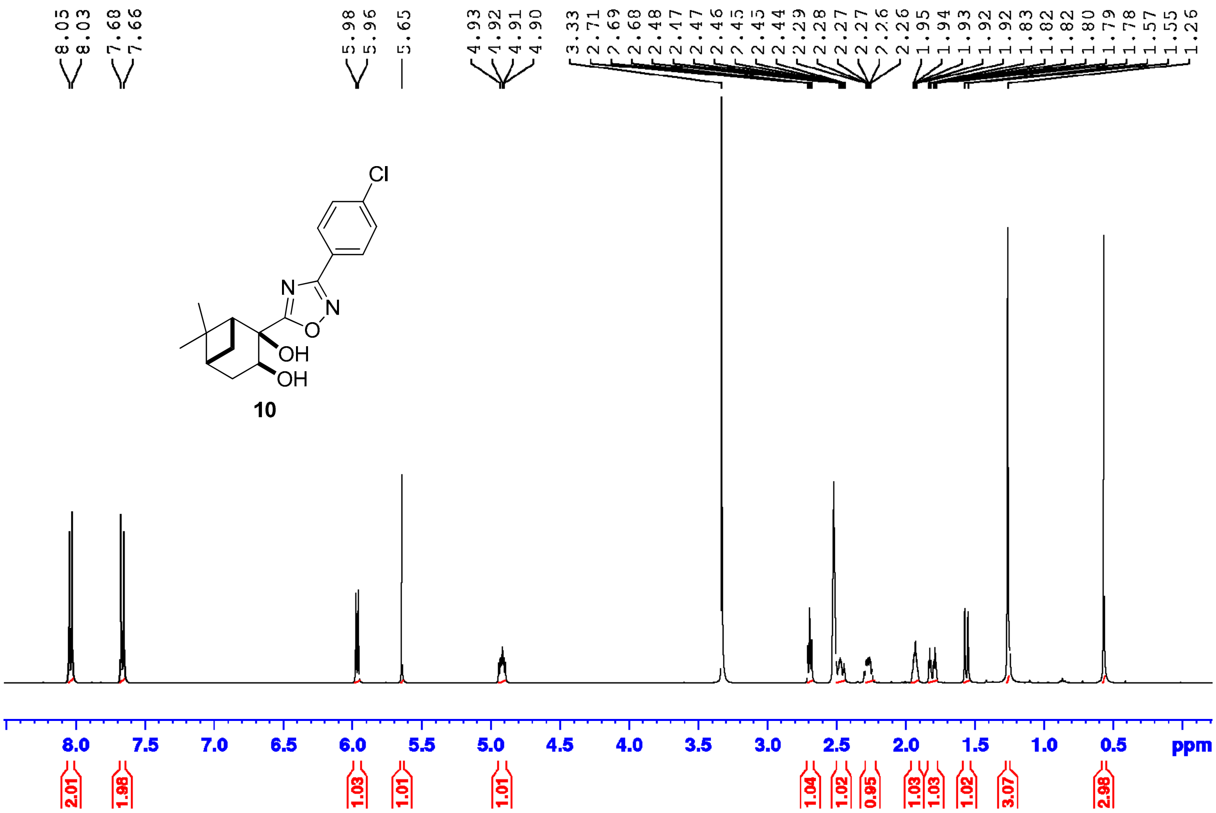

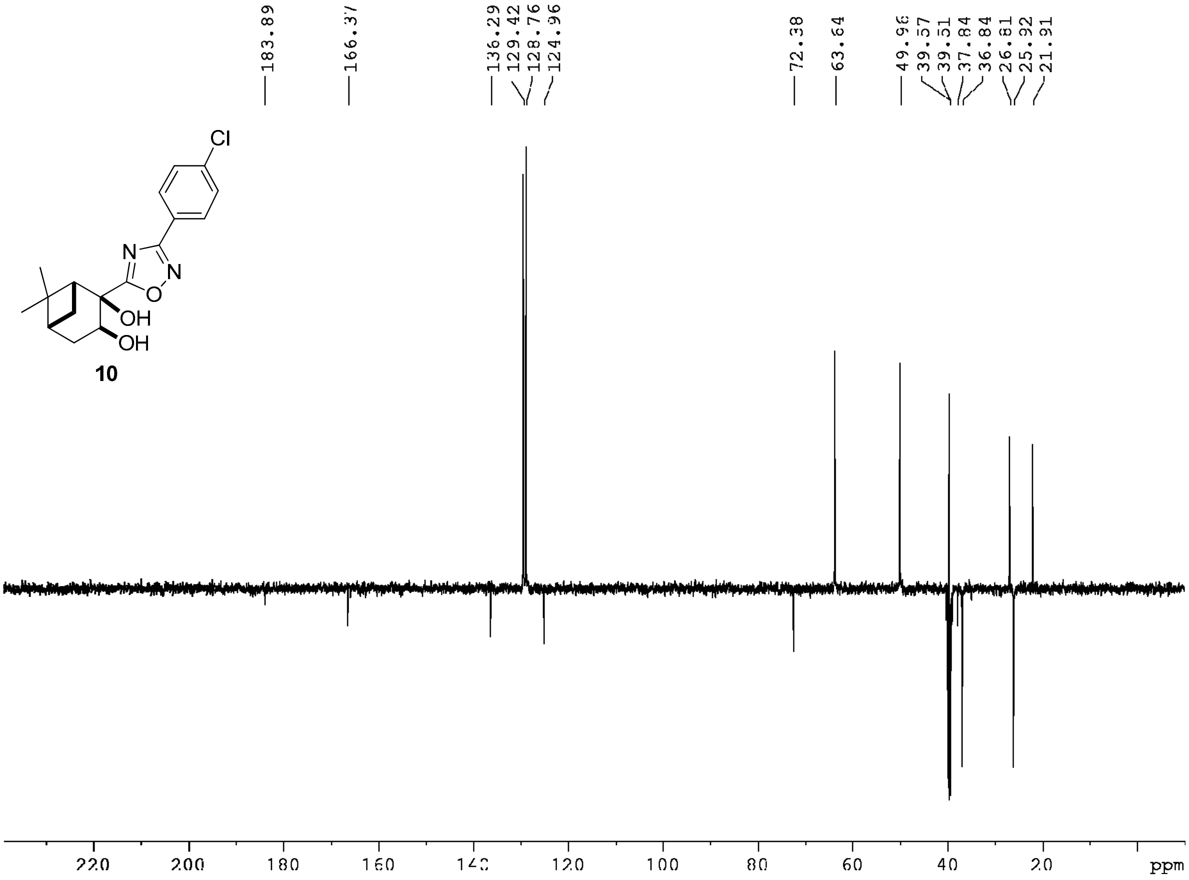

^{1}H- and ^{13}C-NMR spectrum of *(1R,2R,3S,5R)-2-(3-(4-chlorophenyl)-1,2,4-oxadiazol-5-yl)-6,6-dimethylbicyclo[3.1.1]heptane-2,3-diol* (**10**)

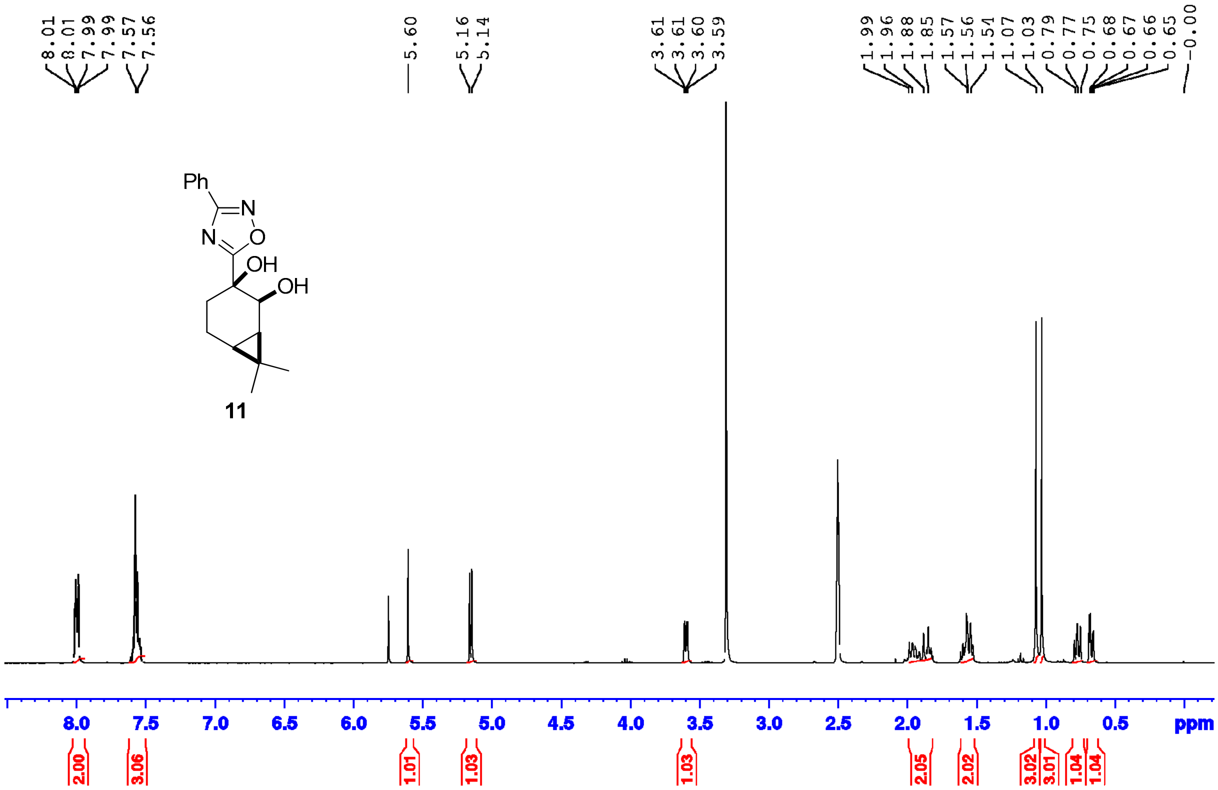

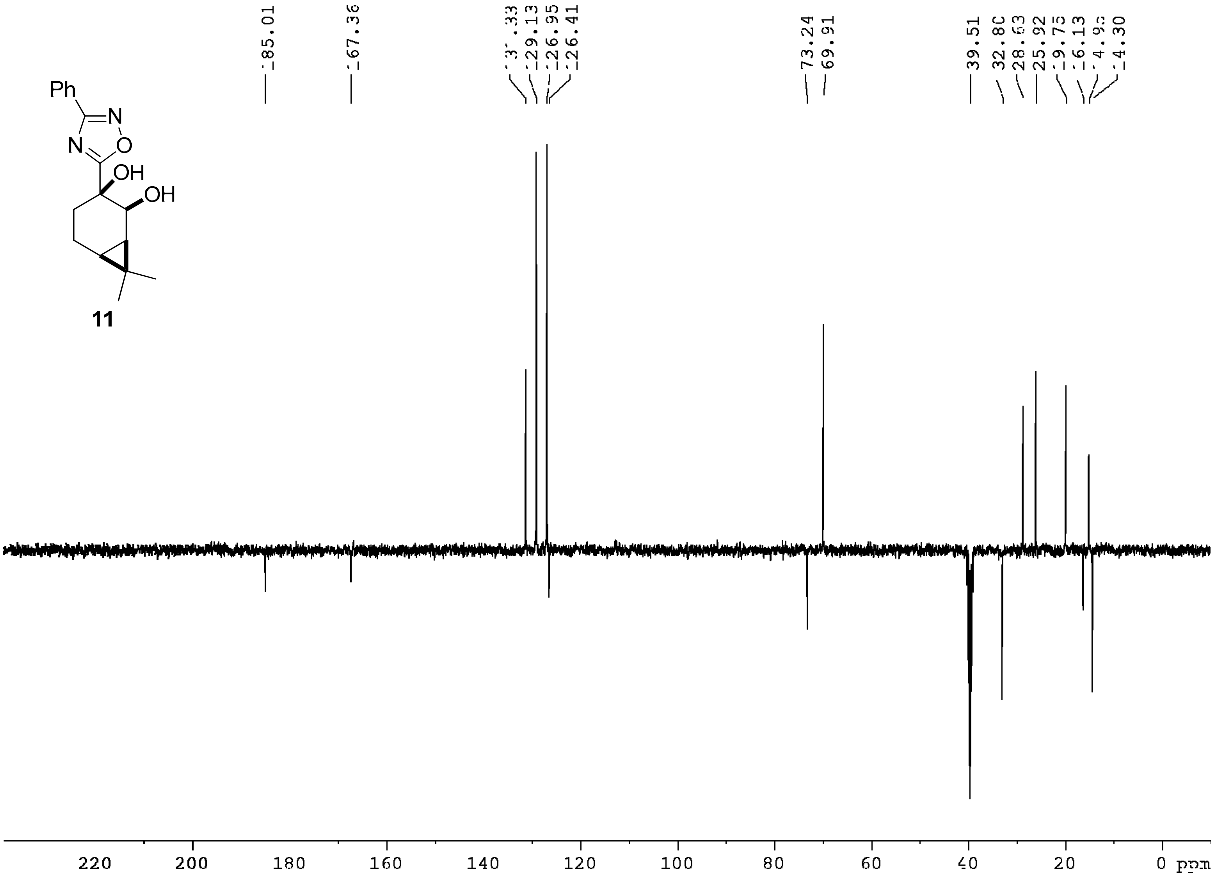

^{1}H- and ^{13}C-NMR spectrum of *(1R,2S,3R,6S)-7,7-dimethyl-3-(3-phenyl-1,2,4-oxadiazol-5-yl)bicyclo[4.1.0]heptane-2,3-diol* (**11**)

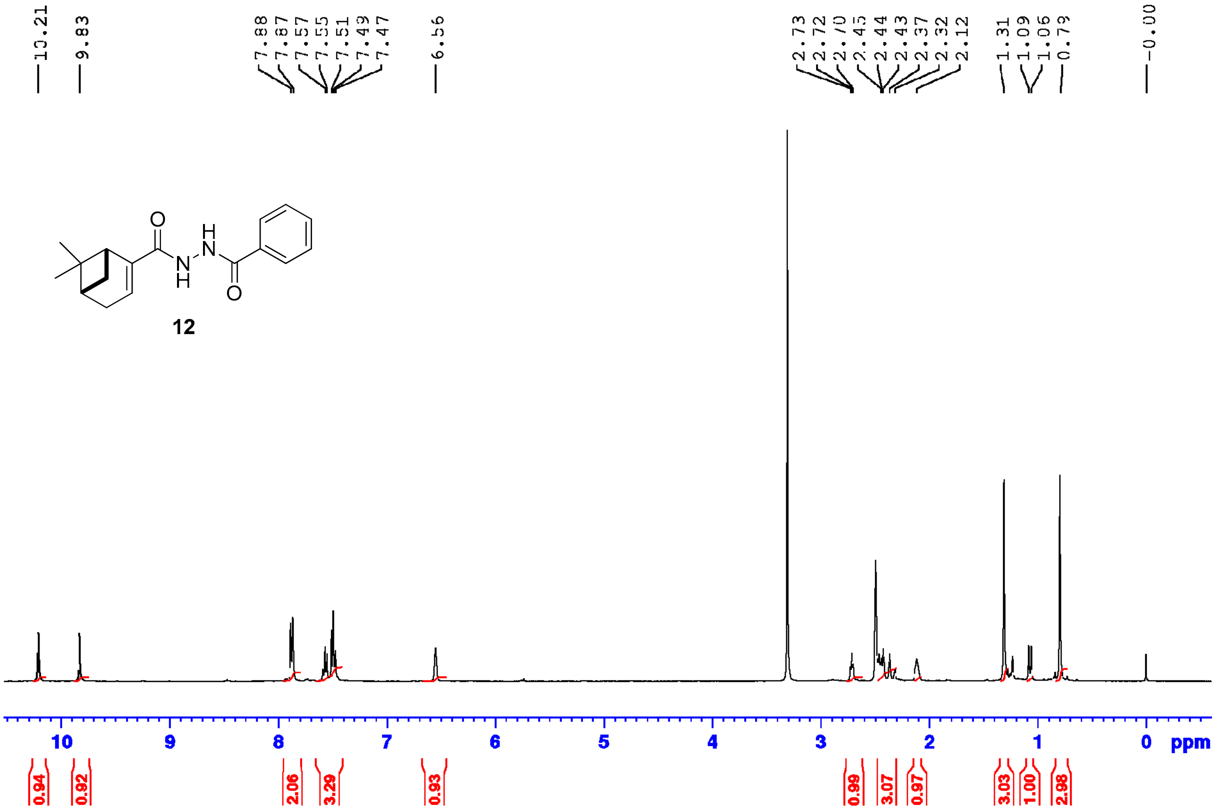

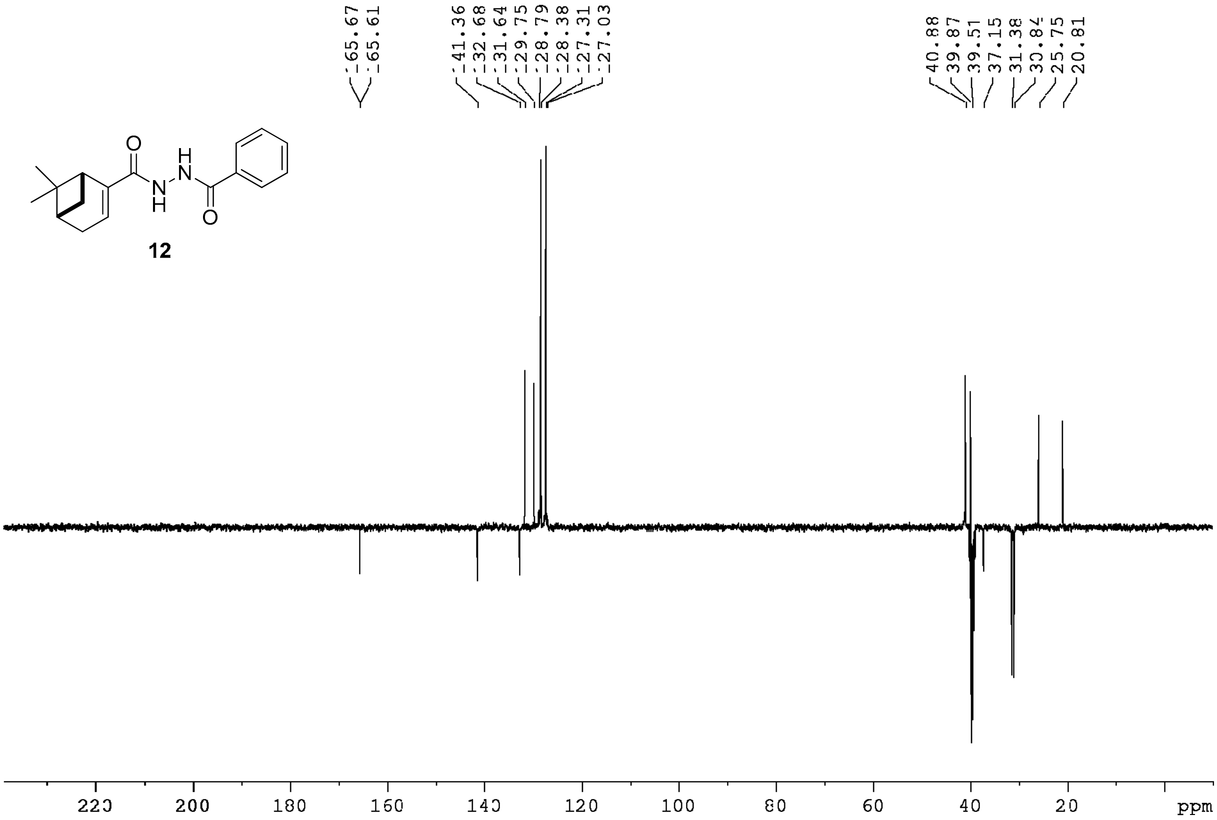

^{1}H- and ^{13}C-NMR spectrum of *(1R,5S)-N'-benzoyl-6,6-dimethylbicyclo[3.1.1]hept-2-ene-2-carbohydrazide* (**12**)

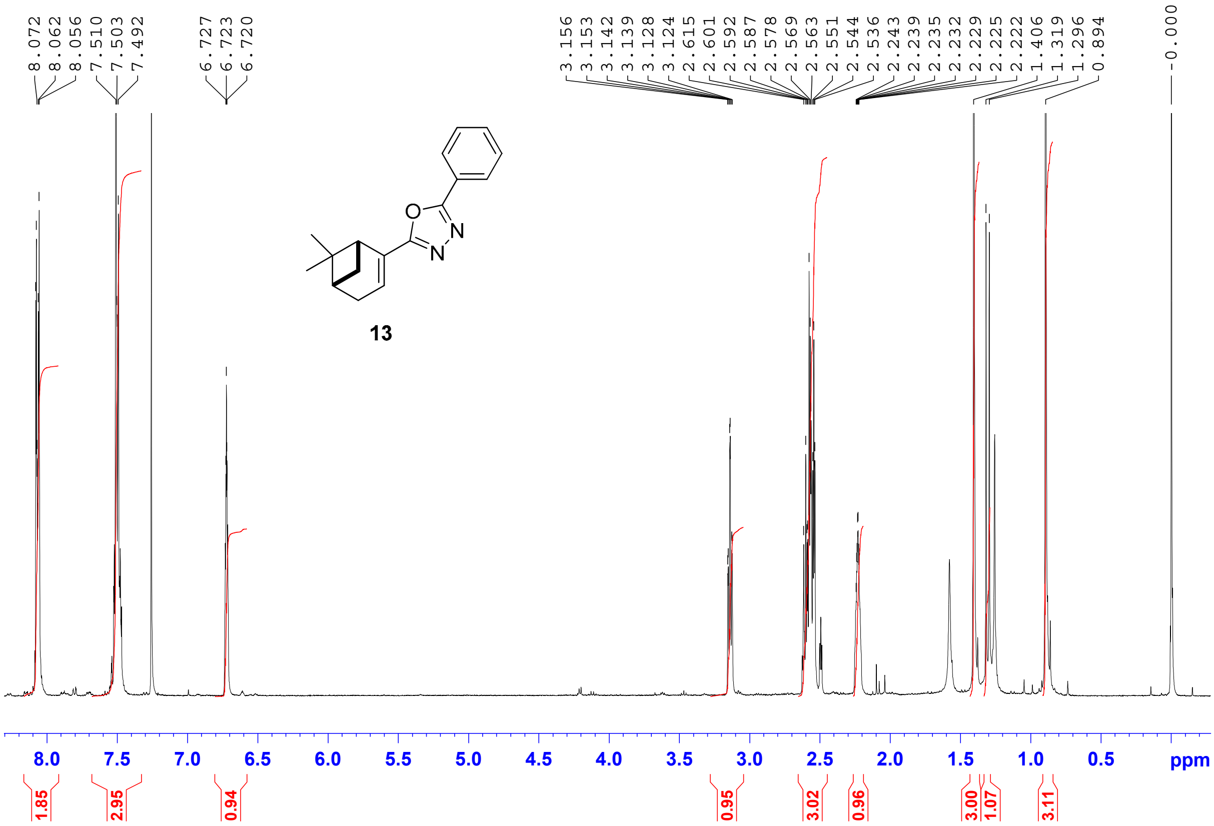

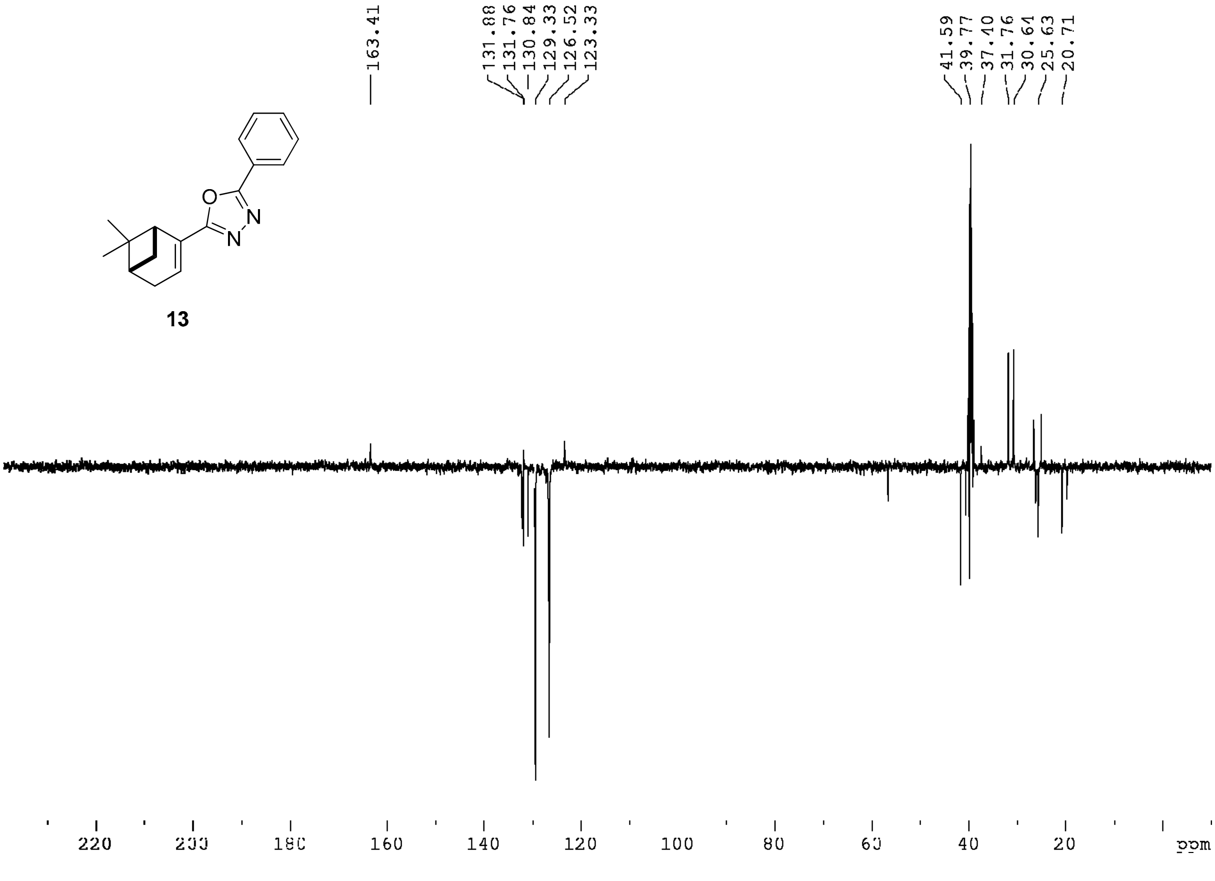

^{1}H- and ^{13}C-NMR spectrum of *2-((1R,5S)-6,6-dimethylbicyclo[3.1.1]hept-2-en-2-yl)-5-phenyl-1,3,4-oxadiazole* (**13**)

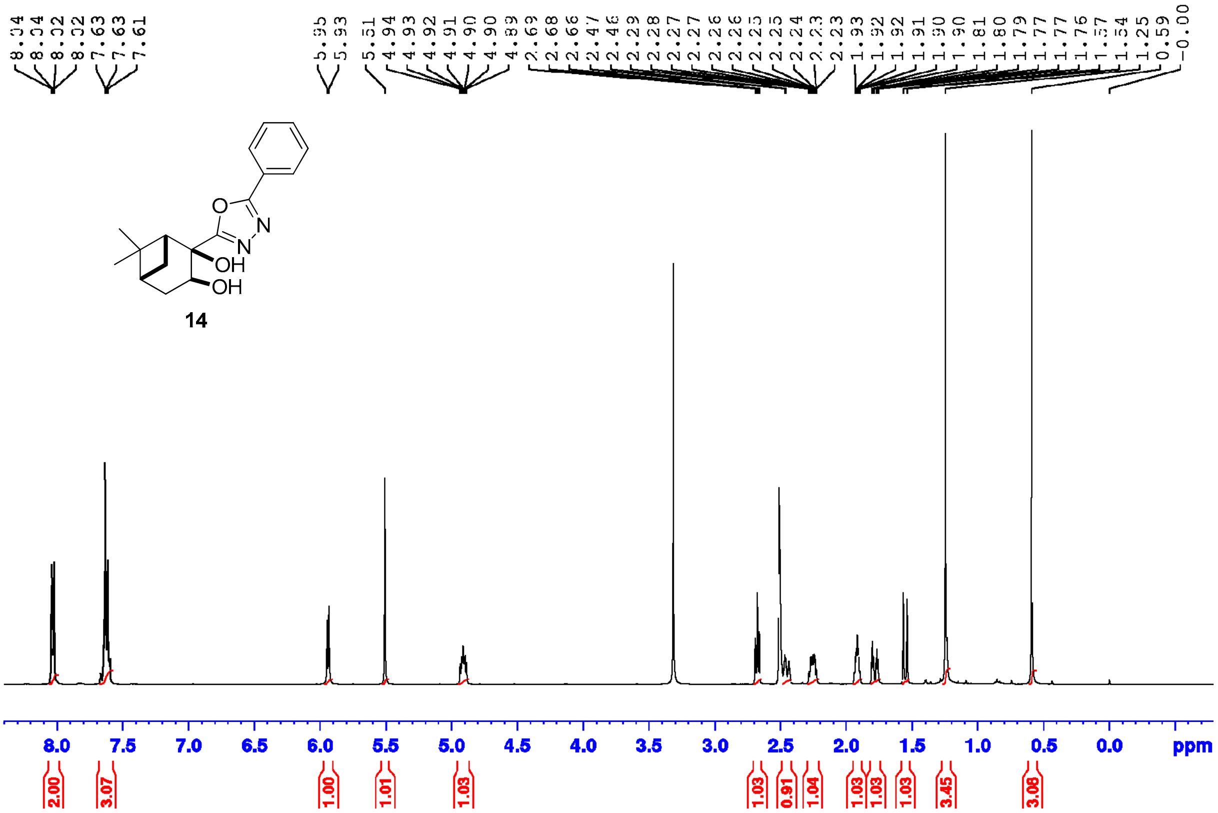

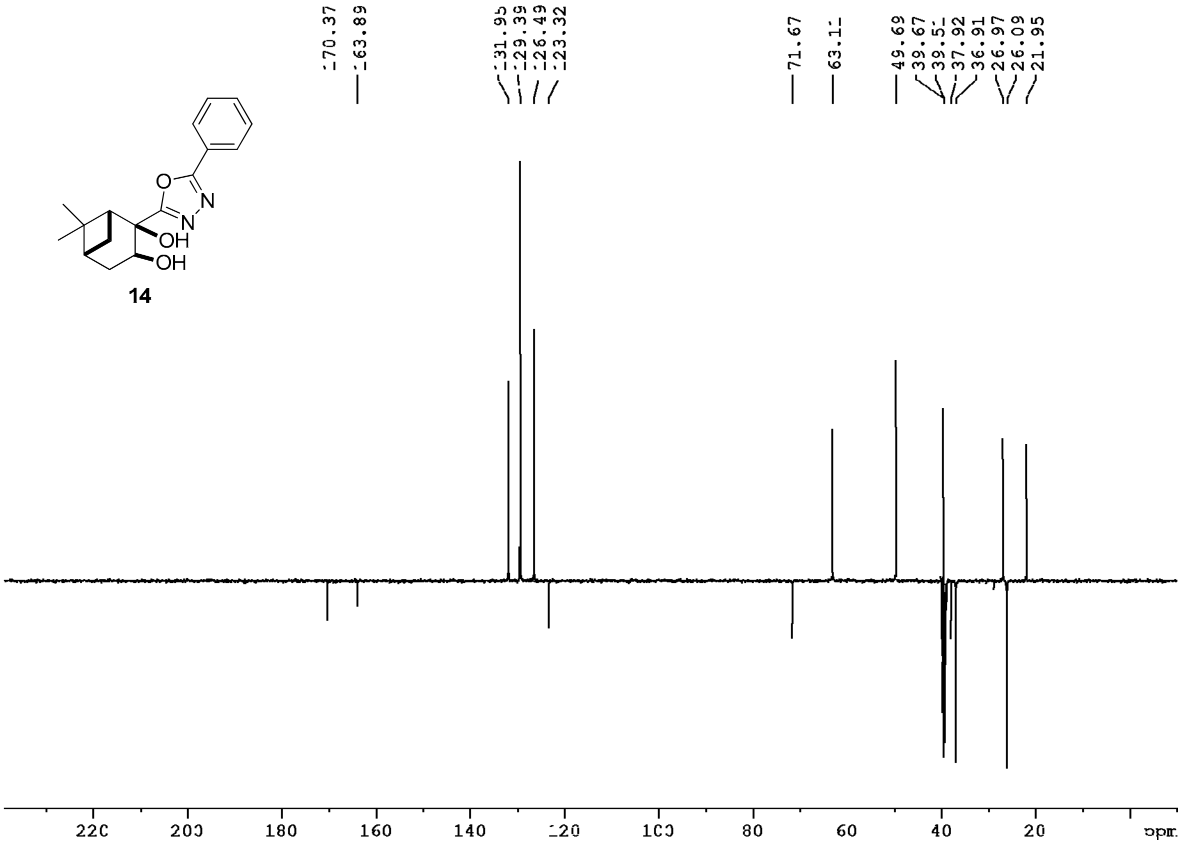

^{1}H- and ^{13}C-NMR spectrum of *(1R,2R,3S,5R)-6,6-dimethyl-2-(5-phenyl-1,3,4-oxadiazol-2-yl)bicyclo[3.1.1]heptane-2,3-diol* (**14**)

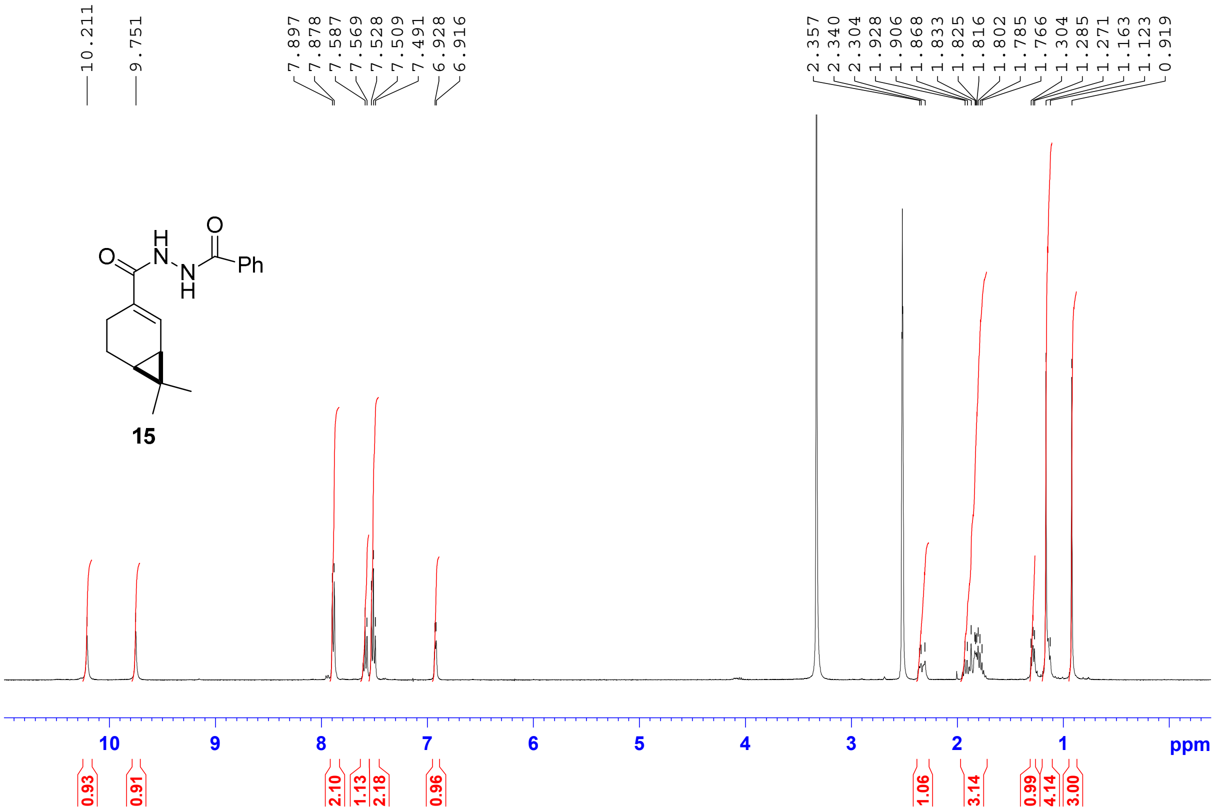

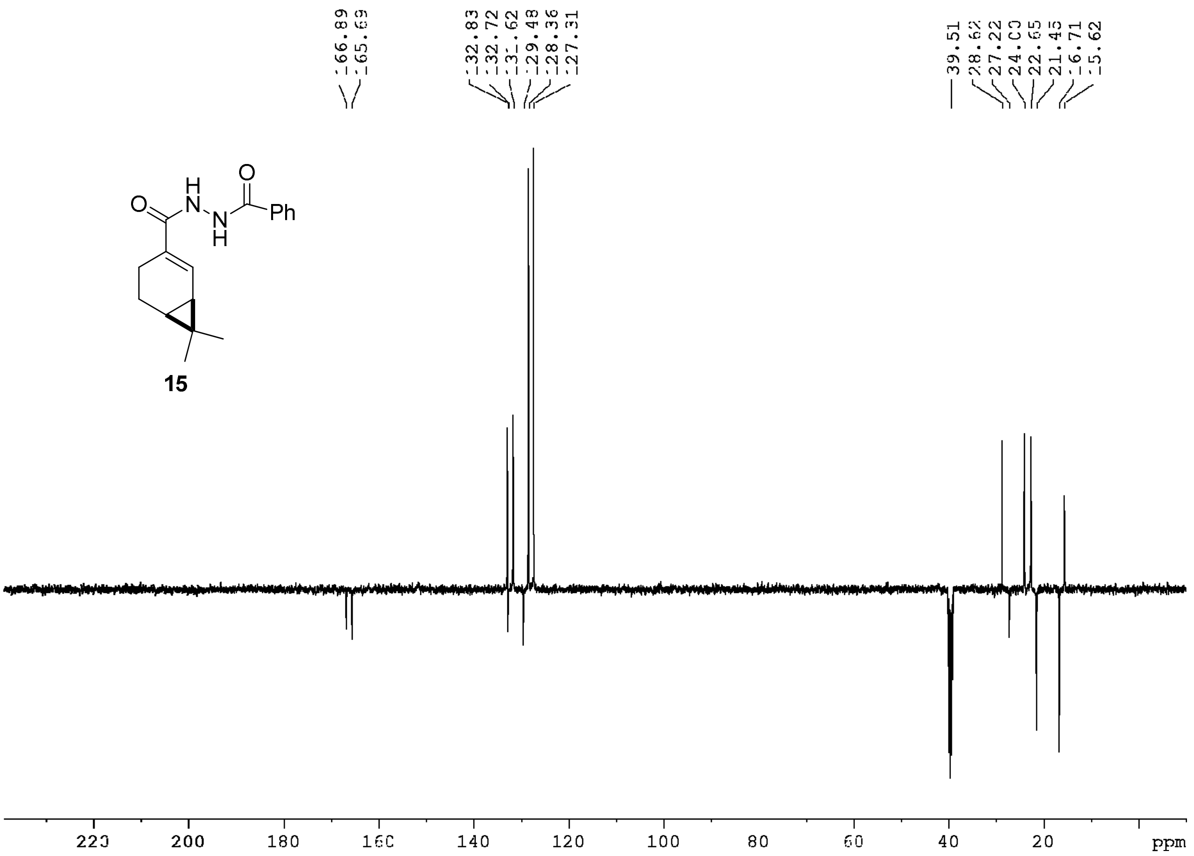

^{1}H- and ^{13}C-NMR spectrum of *(1R,6S)-N'-benzoyl-7,7-dimethylbicyclo[4.1.0]hept-2-ene-3-carbohydrazide* (**15**)

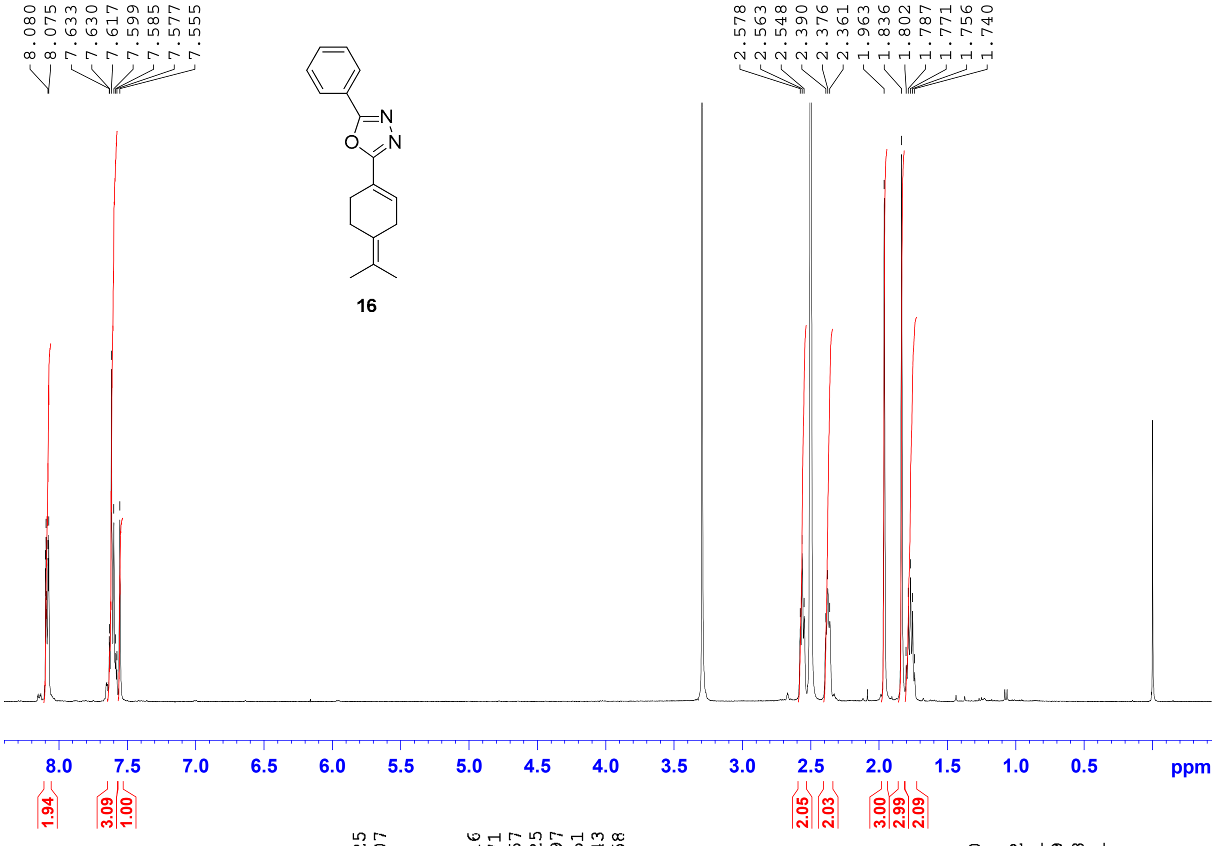

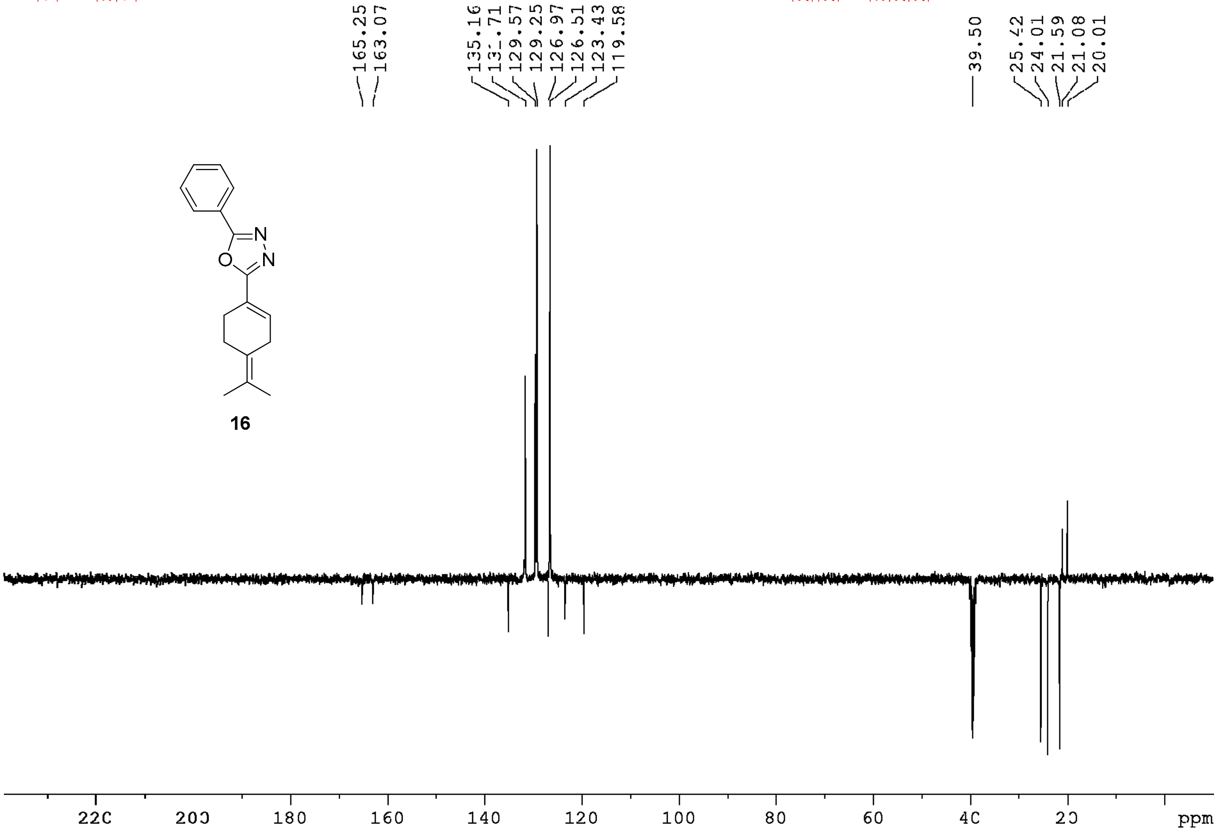

^{1}H- and ^{13}C-NMR spectrum of *2-phenyl-5-(4-(propan-2-ylidene)cyclohex-1-en-1-yl)-1,3,4-oxadiazole* (**16**)

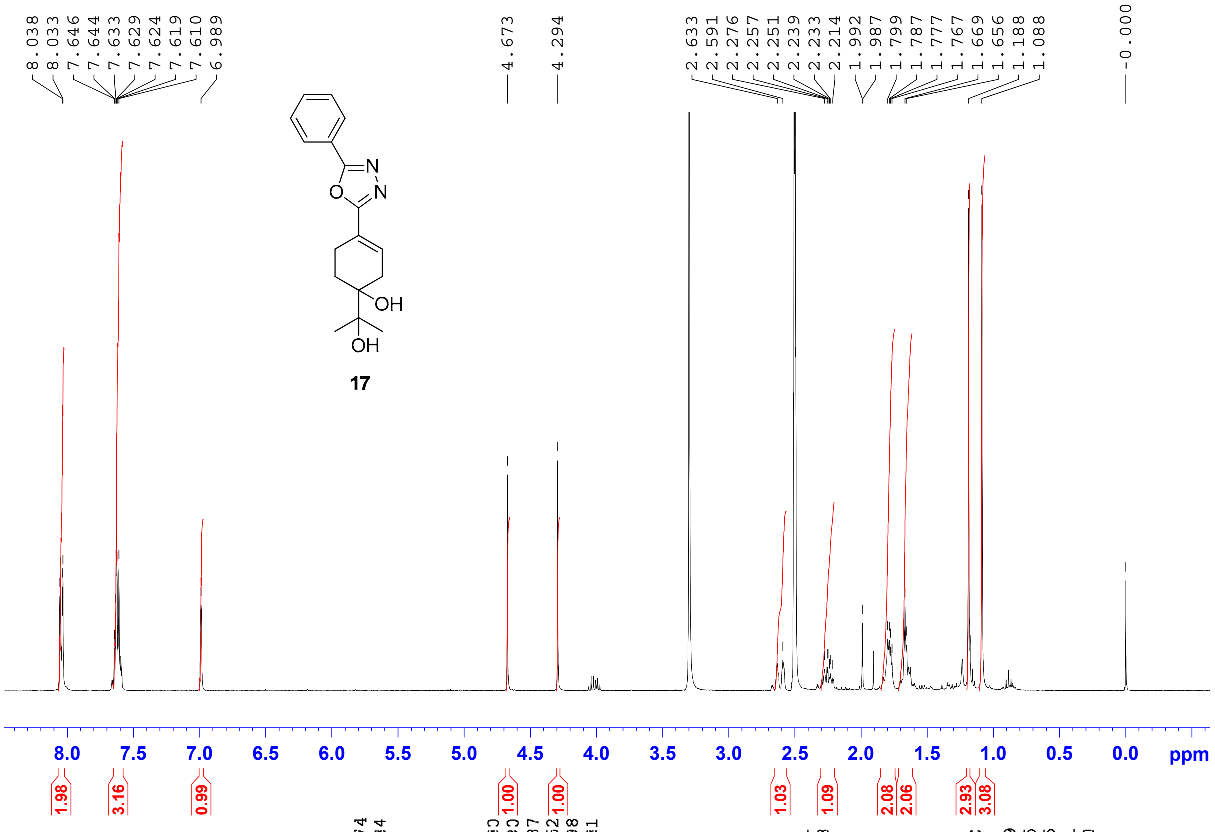

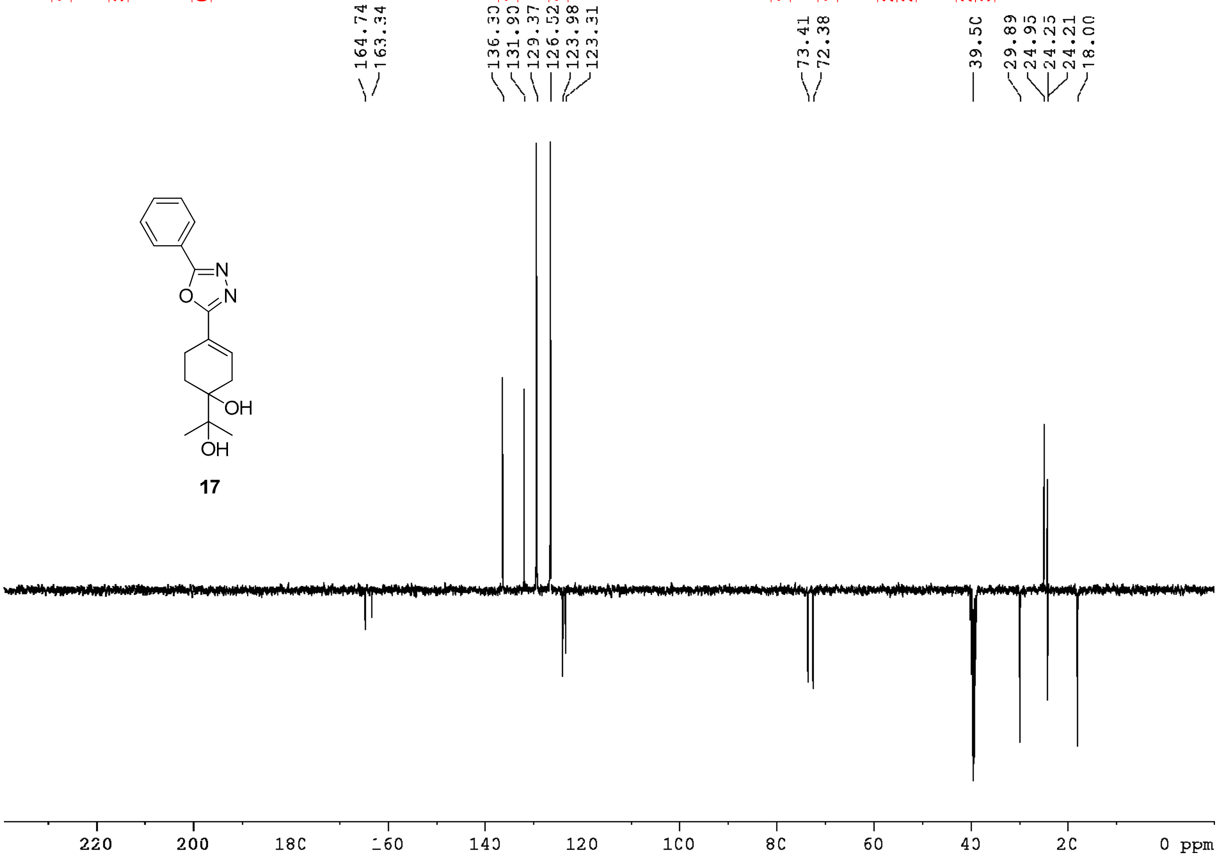

^{1}H- and ^{13}C-NMR spectrum of *1-(2-hydroxypropan-2-yl)-4-(5-phenyl-1,3,4-oxadiazol-2-yl)cyclohex-3-enol* (**17**)
